# Oocyte lipid damage from maternal ageing marks neurodevelopment in mouse offspring

**DOI:** 10.1093/hropen/hoag096

**Published:** 2026-10-19

**Authors:** Hafsa Gulzar, Richard Musson, Joanna Depciuch, Łukasz Gąsior, Simona Bisogno, Michał Sarna, Maria Florencia Heber, Julia Skałka, Diego Alejandro Molina, Michał Kiełbus, Anna Bodzon-Kulakowska, Piotr Suder, Lulu Alluhaibi, Alexey Maximenko, Grażyna E Ptak

**Affiliations:** Malopolska Centre of Biotechnology, Jagiellonian University, Kraków, Poland; Doctoral School of Exact and Natural Sciences, Jagiellonian University, Kraków, Poland; Malopolska Centre of Biotechnology, Jagiellonian University, Kraków, Poland; Doctoral School of Exact and Natural Sciences, Jagiellonian University, Kraków, Poland; Institute of Nuclear Physics, Polish Academy of Sciences, Kraków, Poland; Malopolska Centre of Biotechnology, Jagiellonian University, Kraków, Poland; Malopolska Centre of Biotechnology, Jagiellonian University, Kraków, Poland; Faculty of Biochemistry, Biophysics and Biotechnology, Jagiellonian University, Kraków, Poland; Malopolska Centre of Biotechnology, Jagiellonian University, Kraków, Poland; Malopolska Centre of Biotechnology, Jagiellonian University, Kraków, Poland; Malopolska Centre of Biotechnology, Jagiellonian University, Kraków, Poland; Doctoral School of Exact and Natural Sciences, Jagiellonian University, Kraków, Poland; Department of Biochemistry and Molecular Biology, Medical University of Lublin, Lublin, Poland; Department of Analytical Chemistry and Biochemistry, AGH University of Krakow, Kraków, Poland; Department of Analytical Chemistry and Biochemistry, AGH University of Krakow, Kraków, Poland; SOLARIS National Synchrotron Radiation Centre, Jagiellonian University, Kraków, Poland; SOLARIS National Synchrotron Radiation Centre, Jagiellonian University, Kraków, Poland; Malopolska Centre of Biotechnology, Jagiellonian University, Kraków, Poland

**Keywords:** advanced maternal age, oocyte lipidome, neurodevelopment, embryo transfer, intergenerational persistence, brain lipids, lipid homeostasis, mouse model

## Abstract

**STUDY QUESTION:**

Is advanced maternal age (AMA) associated with lipid dysregulation in oocytes that correlates with the intergenerational persistence of altered lipid homeostasis and neurodevelopmental pathway signatures in downstream offspring?

**SUMMARY ANSWER:**

Lipid profile alterations characterized in AMA oocytes are associated with distinct lipidomic and spectral variations in the foetal brain and yolk sac, displaying intergenerational persistence that extends up to the fourth generation (F4), independently of the gestational uterine environment.

**WHAT IS KNOWN ALREADY:**

AMA is a recognized prenatal risk factor for neurodevelopmental conditions in offspring. However, whether early-life oocyte lipid variations correlate with altered lipid profiles and molecular signatures in the developing brains of subsequent generations remain uncharacterized.

**STUDY DESIGN, SIZE, DURATION:**

This controlled animal model study compared oocytes, blastocysts, and foetuses from AMA mice (8–16 months old) to those from young maternal age (YMA, 3–4 months old) controls and featured an embryo transfer (ET) of AMA blastocysts into young surrogates and longitudinal, lineage-specific tracking up to the F4.

**PARTICIPANTS/MATERIALS, SETTING, METHODS:**

Complementary approaches, including liquid chromatography-mass spectrometry (LC-MS) proteomics and lipidomics, Matrix-Assisted Laser Desorption/Ionization–Tandem Time-of-Flight mass spectrometry (MALDI-TOF/TOF MS), RNA sequencing, atomic force microscopy, Coherent Anti-Stokes Raman Scattering (CARS) chemical imaging microscopy, Raman and Fourier transform infrared (FTIR) spectroscopies, and histology were conducted on single oocytes, blastocysts, and foetal structures (brain and yolk sac) from AMA and YMA mice. ET was utilized to decouple the oocyte-derived phenotype from the effects of an ageing maternal uterine environment.

**MAIN RESULTS AND THE ROLE OF CHANCE:**

AMA oocytes and preimplantation blastocysts exhibited significant lipid droplet and lipid membrane alterations, a higher relative absorbance of intracellular lipid bands, and altered expression of lipid-regulatory transcripts, corresponding to early embryonic pathway shifts. Following the ET of AMA blastocysts to young surrogates (ET AMA group), distinct lipidomic variations and structural alterations were observed in the F1 yolk sac and foetal brains. Furthermore, these lipid-associated spectral and molecular signatures displayed intergenerational persistence, tracking through the yolk sac, brain, and oocytes of the third- (F3) and fourth-generation (F4) descendants derived from the oocyte of AMA progenitors.

**LARGE SCALE DATA:**

The transcriptomic data have been deposited in the NCBI Gene Expression Omnibus (GEO) under accession number GSE349634. Processed transcriptomic, lipidomic, and proteomic datasets, together with the shared transcript–protein analysis, have been deposited in RODBUK under DOI https://doi.org/10.57903/UJ/LVQ7FU. All datasets are publicly available.

**LIMITATIONS, REASONS FOR CAUTION:**

This study was conducted exclusively in a mouse model. Given species-specific variations in baseline oocyte lipid content and developmental kinetics, these findings require cautious interpretation before extrapolation to human clinical contexts. Additionally, the exact molecular and epigenetic mechanisms underlying the intergenerational persistence of these lipid signatures warrant further characterization.

**WIDER IMPLICATIONS OF THE FINDINGS:**

These findings demonstrate an association between early-life oocyte lipid changes and downstream pathway-level molecular signatures of neurodevelopmental alterations. By establishing an intergenerational pathway that persists independently of the maternal uterine environment, this work introduces a novel framework linking metabolic programming at conception to neurodevelopmental outcomes, offering new directions for reproductive medicine and translational neuroscience.

**FUNDING:**

This research was funded by National Science Centre of Poland Grants 2021/41/B/NZ3/03507 and 2019/35/B/NZ4/03547 (to G.E.P.), 2022/44/C/NZ4/00076 (to M.F.H.), and 2019/35/N/NZ3/03213 (to Ł.G.), and the program ‘Excellence initiative–research university’ for the AGH University of Krakow (A.B.-K.). Proteomics was supported by the subsidy no 16.16.160.557 of the Polish Ministry of Science and Higher Education (to P.S.). Raman analysis at the National Synchrotron Radiation Centre SOLARIS was supported by the Ministry of Science and Higher Education, Poland, under contract no. 1/SOL/2021/2.

**DISCLOSURES:**

The authors declare no competing interests.

WHAT DOES THIS MEAN FOR PATIENTS? As women get older, it becomes more difficult to get pregnant, and there is a statistically higher probability of their children having conditions that affect how their brains grow and function. While scientists are still trying to understand why this happens, our research has uncovered a new clue: ageing is strongly associated with an overload and alterations of fat molecules (lipids) inside a mother’s eggs (oocytes). Until now, it has remained uncharacterized whether these early-life fat variations in the oocyte correlate with the baby’s brain development.

In this study, we looked at how maternal age relates to fat balance in mouse oocytes and growing embryos, and how this tracks with the developing brain. We found that oocytes from older mothers have distinct fat imbalances and altered fat structures. By transferring these embryos into young surrogate mothers, we showed that these fat variations strongly correlate with alterations in brain development in the offspring, occurring independently of the ageing womb. Remarkably, these exact fat imbalances in the brain and oocytes displayed an intergenerational persistence, continuing to appear up to the fourth generation of descendants.

For patients and families, these findings provide an interesting new perspective in reproductive medicine. They demonstrate for the first time a link between the lipid profile of an oocyte from an older mother and a lasting molecular lipid imprint that correlates with brain development pathways across future generations.

## Introduction

The aetiology of neurodevelopmental (ND) disorders is complex, reflecting their multifactorial origin in which environmental and polygenic components play a role in the vast majority of patients. This complexity makes it difficult to identify the underlying pathophysiological mechanisms. One prenatal factor consistently associated with ND disorders is the age of the parents, especially advanced maternal age (AMA) (Byrne *et al.*, 2003; Kim *et al.*, 2019; Gao *et al.*, 2023). The ageing process is associated with elevated reactive oxygen species (ROS) production and a decline in antioxidant defences, thus resulting in oxidative stress (OS) in germinal cells and damage to cellular lipids. Lipids are especially prone to oxidation, more so than DNA and proteins, due to the carbon–carbon double bonds present in unsaturated fatty acids (FAs). In females, ageing-related oxidative changes in oocyte lipids, characterized by altered membrane morphology, changes in lipid droplet (LD) profile, and accumulation of lipid peroxidation (LPO) products, have been recently documented in humans and mice (Mihalas *et al.*, 2017a, 2018; Bisogno *et al.*, 2024; Gulzar *et al.*, 2026). As LDs in the oocyte store essential resources for embryonic signalling and membrane assembly (Arena *et al.*, 2021), age-related disruption of lipid homeostasis may have consequences beyond the oocyte itself. Altered lipid composition has been linked to ND disorders, particularly those involving synaptic dysfunction (synaptopathies) and ageing (Liu *et al.*, 2015; Marschallinger *et al.*, 2020; Yu *et al.*, 2020; Yap *et al.*, 2023; Tüscher *et al.*, 2024). Importantly, embryonic neurulation starts well before placental nutrient transfer is fully established, and early brain development relies on microglia derived from the embryonic yolk sac (YS) (Paolicelli *et al.*, 2011). Consequently, if lipid reserves in the embryo are compromised by OS, as may occur in AMA, neurodevelopment, and especially neurosignalling processes, may be adversely affected.

In this article, we evaluate how lipid homeostasis is disrupted in AMA oocytes, blastocysts, and foetuses. To isolate the effect of oocyte quality from suboptimal uterine conditions of aged females, blastocysts derived from aged mothers (F0) were transferred to the uteri of young surrogate females via embryo transfer (ET) procedure to produce the F1 generation (ET AMA group). In one cohort, pregnancies were terminated at embryonic Day 16 or 19 (E16 or E19) to collect foetuses for analysis of ND-relevant organs: brain and YS. The remaining pregnancies were carried to term, and F1 offspring were subsequently mated with young partners to produce successive generations. We tracked these descendants for intergenerational persistence, producing F3 males for adult brain analysis, F3 females for oocyte analysis (ET AMA F3), and F4 embryos for brain and YS analysis (ET AMA F4) (Supplementary Fig. S1).

## Materials and methods

All chemicals, unless otherwise indicated, were obtained from Sigma-Aldrich (St. Louis, MO, USA).

### Animal studies

All procedures complied with the European Community Regulation 86/609 and adhered to the Polish Governmental Act for Animal Care. Ethical approval was granted by the II Local Ethical Commission of Kraków (permission no. 123/2018 and 329/2021). Animal experiments were conducted using C57BL/6 as oocyte and embryo donors and C57Bl/6 × CBA as embryo recipients (hybrid mice are superior in fostering mice), with the AMA group consisting of 8–16 month-old females and the young maternal age (YMA) group comprising 3–4 month-old females. Male C57BL/6 mice used for mating were 3 months old. Animals were housed under temperature-controlled conditions (22°C) with a 12-h light–dark cycle and were provided food and water *ad libitum*.

### GV and MII stage oocyte collection

Germinal vesicle (GV)-stage oocytes were retrieved from the ovaries of AMA and YMA females. Ovaries were excised, and surrounding adipose tissue was removed. Dissection was performed using a 26-gauge needle, enabling the isolation of GV-stage oocytes encased by cumulus cells, which were subsequently collected in M2 medium. The surrounding cumulus cells were removed through hyaluronidase treatment (100 IU/ml) followed by washing in M2 medium. For metaphase II (MII)-stage oocytes, AMA and YMA females were subjected to superovulation: mice received an intraperitoneal (i.p.) injection of 5 IU of pregnant mare serum gonadotropin, followed 48 h later by an i.p. injection of 5 IU hCG. Oocytes at the MII stage were harvested from the oviductal ampullae 14 h post-hCG administration and maintained in M2 medium during processing.

### Embryo collection and transfer

AMA and YMA females were paired with stud males overnight, and vaginal plugs were checked the following morning to confirm mating. Part of plug-positive females were allowed to continue the pregnancies (natural pregnancy (NP) groups: NP AMA and NP YMA). Another part of plug-positive females was euthanized by cervical dislocation 4 days post-coitum to collect embryos. Embryos were recovered by flushing each uterine horn with 1 ml of M2 medium. Part of blastocysts was transferred to 3-month-old CBA × C57BL/6 pseudo-pregnant females (ET AMA and ET YMA groups), as described previously (Arena *et al.*, 2021). One cohort of pregnancies were allowed to go to term to allow the production of the third generation of AMA-derived mice. Another cohort of pregnant females were ethically sacrificed either at E16 or E19 to collect foetal organs for analysis (Supplementary Fig. S1).

### Production of the third generation of AMA-derived offspring

Day 4 embryos from AMA and YMA C57BL/6 donor mice were transferred to 3-month-old CBA × C57BL/6 female recipients as described previously (Arena *et al.*, 2021). Recipients were allowed to deliver spontaneously. Resulting offspring were subjected to a transgenerational study whereby, upon reaching 3 months of age, they were mated with young control C57BL/6 mice, and offspring were tracked for three generations, creating ET AMA F3 and ET YMA F3 experimental groups. F3 mice were sacrificed for tissue collection at 5 months of age. Female F3 mice were sacrificed at E9 following mating with C57BL/6 males to obtain F4 embryonic tissues.

### Brain and YS collection

For adult brain collection, animals were ethically sacrificed by decapitation. A midline incision was made in the skin. Remaining muscles and skin were removed to reveal the skull. After cutting the optic nerve behind each eyeball, a cut was made in the skull between the nasal and frontal areas. Using Pean’s forceps or spatula, all bones were peeled off starting from the occipital area. The brains were extracted, weighed, and immediately placed in an ice-cold Petri dish, where the brain (either entire organ or the region of interest, such as the cerebellum or prefrontal cortex) was dissected, snap-frozen in liquid nitrogen, and stored at −80°C. For foetal brain and YS collection, pregnant females from all groups (ET AMA, ET YMA, NP AMA, and NP YMA) were sacrificed by cervical dislocation. A midline incision of the ventral skin and abdominal wall muscles was performed to expose and remove the uterus containing embryos. After the removal of remaining uterine tissue, each embryo was carefully removed out of its YS and ethically euthanized. The foetal brain and YS were isolated using curved forceps and stored at −80°C. Part of foetuses were left intact and processed for histological analysis.

### Raman spectroscopy

Raman spectroscopy was performed on individual GV-stage mouse oocytes (AMA, YMA) using the MonoVista CRS3 500 system at the complementary techniques section at the ASTRA beamline at the SOLARIS Synchrotron (Krakow, Poland). Measurements were conducted with a 10× objective lens and a 532 nm laser, with the laser power calibrated for optimal signal-to-noise ratio. The exposure time was set to 10 s per accumulation. Oocytes were placed on calcium fluoride Raman-grade glass substrates for spectral acquisition. Prior to measurement, samples were precisely focused using the integrated Raman microscope to ensure accuracy. The resulting Raman spectra were processed using OPUS software, which involved baseline correction and normalization to standardize spectral data for subsequent analysis. Raman spectra were analysed with a particular focus on characteristic Raman shifts corresponding to specific biochemical compounds: acetylcarnitine (1184 cm^−1^), nicotinamide adenine dinucleotide (NAD^+^) (1330 cm^−1^), fumarate (1401 cm^−1^), acetyl-CoA (1620 cm^−1^), S-adenosyl methionine (SAM) (2920 cm^−1^), cholesterol (Chol) (2930 cm^−1^), and FAs (∼3010 cm^−1^). Statistical analysis was performed based on the intensities of these specific Raman peaks. Differences between groups were evaluated using one-way ANOVA and Tukey’s *post hoc* test (significance threshold *P *< 0.05), conducted in PAST software version 4.17 (Hammer *et al.*, 2001). In addition, principal component analysis (PCA) was applied to the 800–1800 cm^−1^ range to identify global spectral variations and clustering patterns among the groups.

### FTIR spectroscopy

The Fourier transform infrared (FTIR) spectra of samples were obtained using a Nicolet iS50 FTIR spectrometer (Thermo Scientific, Waltham, MA, USA), using the attenuated total reflectance technique, as described previously (Gulzar *et al.*, 2026). Individual adult medial prefrontal cortex samples were homogenized in 50 µl of PBS using a manual pestle. To obtain FTIR spectra, 2 µl of PBS solution containing an individual sample was dropped onto the CaF_2_ crystal. GV oocytes, blastocysts, E9 embryonic brains, and YSs were placed onto the CaF_2_ crystal individually in a small drop of PBS without any processing. All samples were measured in the range of selected infrared radiation between 400 and 4000 cm^−1^ with the spectral resolution 4 cm^−1^ and 324 scans. The resulting spectra were normalized using a min.–max. normalization by means of the OPUS 7.0 software. Spectral analysis focused on absorbance values corresponding to biochemical compounds of interest, including: phosphatidylinositol (PI: 576 cm^−1^), phosphatidylcholine (PC: 719 cm^−1^), phosphatidylserine (PS: 733 cm^−1^), sphingomyelin (SM: 875 cm^−1^), ceramides (Cer: 898 cm^−1^), phosphatidylethanolamine (PE: 1038 cm^−1^), alkyl phosphatidylcholine (PC O–: 1120 cm^−1^), fumarate (1207 cm^−1^), SAM (1330 cm^−1^), NAD^+^ (1580 cm^−1^), α-ketoglutarate (α-KG: 1750 cm^−1^), Chol (2930 cm^−1^), and polyunsaturated FAs (PUFA: ∼3000 cm^−1^). Similarly, PCA was performed in the 800–1800 cm^−1^ range to evaluate overall biochemical variance between oocyte groups. Furthermore, the membrane fluidity was assessed using the intensity ratio of the CH_2_ asymmetric (2909 cm^−1^) and symmetric (2850 cm^−1^) stretching vibrations, as this ratio reflects the degree of lipid chain disorder. Changes in the protein secondary structure were determined from the ratio of absorbance at 1650 cm^−1^ (α-helix) and 1620 cm^−1^ (β-sheet), which correspond to characteristic amide I band components, providing insight into conformational alterations in proteins. Statistical analysis was conducted based on the absorbance intensities of these peaks using one-way ANOVA and Tukey’s *post hoc* test (*P *< 0.05, PAST 4.17 software).

### Zeta potential

The ζ-potential distribution of AMA and YMA GV oocytes was assessed using the microelectrophoretic method with the Zetasizer Ultra system (Malvern Instruments, Malvern, UK). Measurements were conducted employing the Smoluchowski model for calculating ζ-potential. For both groups, three independent runs were performed, each run containing a pool of 10 GV oocytes, with at least 100 measurements per run to ensure robustness. For *Z*-potential, measurements were carried out in a 0.9% NaCl solution at 25°C. Statistical analysis was performed in PAST.4.17 software; one-way ANOVA followed by Tukey’s *post hoc* test (*P *< 0.05).

### Atomic force microscopy

Measurements were performed with a BioScope Catalyst atomic force microscope (Bruker: Billerica, MA, USA) coupled with an Axio Observer Z1 inverted optical microscope from Zeiss (Oberkochen, Germany). Before the analysis, the zona pellucida was removed from GV-stage oocytes. During the analysis, zona-free GV oocytes were maintained in M2 medium at 37°C. Elasticity measurements of the oocytes were made by employing force spectroscopy mode that consists of measuring force–displacement curves as described previously (Sarna *et al.*, 2017). In brief, before measuring an individual oocyte, the atomic force microscopy (AFM) probe was positioned on top of the zona-free oocyte and aligned using optical microscopy preview. Once aligned, force curves from a grid of 10 × 10 points were collected at a rate of 1 Hz, and 2–3 positions on the oocyte were randomly selected. A soft cantilever with a nominal tip radius of 20 nm and a spring constant of 0.01 N/m (Bruker Probes) was used for analysis (Wiktor *et al.*, 2018).

The obtained force–displacement curves were analysed using the AtomicJ software (Hermanowicz *et al.*, 2014). Statistical analysis was performed using Student’s *t*-test based on data normality (*P *< 0.001).

### MALDI sample preparation and analysis

Sample preparation and analysis were performed as described previously (Bodzon-Kulakowska *et al.*, 2020). Briefly, individual MII oocytes were placed on the indium tin oxide (ITO)-coated glass slides. ITO coating boosts transparency and high-level conductivity in slides. Samples placed on one slide (to minimize the variability) were measured from left to right. For the positive ion mode, a 2,5-dihydroxybenzoic acid (DHB) matrix was prepared at a concentration of 25 mg/ml in methanol–water solution (1:1, v/v); trifluoroacetic acid was added (+0.2% v/v), according to the manufacturer’s recommendation. The matrix was deposited on the samples using a SunCollect device (SunChrom GmbH, Friedrichsdorf, Germany) via the application of 10 layers with a line distance of 2 mm, a speed of 600 mm/min, and with *Z* = 1 mm (nozzle height over the sample). The flow rate of the matrix solution was variable: 10 μl/min for the first layer, 20 μl/min for the second layer, 30 μl/min for the third layer, 40 μl/min for the fourth layer, and 60 μl/min for all other layers. UltrafleXtreme MALDI-TOF/TOF instrument was used (Bruker-Daltonics, Bremen, Germany) for the analysis. Calibration of the spectrometer was performed using the Peptide Calibration Mixture 2 provided by the MS manufacturer. The TOF analyser was set in the reflectron analysis mode to obtain the best resolution. For lipid analysis, the MS spectra acquisition was as follows: scan range: m/z 200–3000 m/z, laser power: 80%, and matrix suppression: off. In a single cycle, two times 400 laser shots were used for spectrum acquisition. Holder’s random walk was limited to 100 μm diameter in a ‘partial sample’ mode, with 20 shots at a raster spot. Fragmentation, based on the LIFT technology for lipid identification, was used. For every MS/MS spectrum acquired, manual selection of the parent ion was done.

A multivariate Partial Least Squares (PLS) regression was used for the analysis of data obtained from the measurement of all oocytes. The number of classes was defined as three, for three different groups (assigned as 1, 0, −1). This results in six permutations, and the permutation giving the best model is presented. Pre-processing of the data involved: summing up channels with the same m/z, data binning resulting in 0.5 m/z data channel separation, data narrowing (640–850 m/z) relevant for PLS regression, and denoising using least-squares polynomial method with three-point span and baseline correction. Each spectrum was normalized to its sum: total ion current method. Kennard–Stone algorithm was used to divide samples into a model dataset (30 samples, 60 spectra) and test dataset (6 samples, 12 spectra). The model dataset was used to find the optimal hyperparameter (LV—Latent Variables) for the final PLS model by application of 30-fold cross validation.

### CARS microscopy

The distribution of LDs in individual MII oocytes was evaluated using coherent anti-stokes Raman scattering (CARS) as described previously (Arena *et al.*, 2021; Bisogno *et al.*, 2024). The multimodal nonlinear microscope used in this study consisted of a Leica DMi8 inverted microscope equipped with the Leica TCS SP8 CARS module and the Leica SP8 confocal module (Leica Microsystems, Wetzlar, Germany). The Leica TCS SP8 CARS uses a tunable pump laser with a tuning range of 780–940 nm, combined with a Stokes laser at 1064 nm, provided by Laser picoEmerald (APE, Berlin, Germany) and integrated with a 750 mW power optical parametric oscillator. The individual oocyte was placed in a Lab-Tek chamber (Thermo Scientific). To detect the LD signal, the optical parametric oscillator’s wavelength was tuned to 816.7 nm to serve as the pump beam, in combination with the 1064 nm Stokes beam to probe the CH_2_ stretch vibration. All signals were detected using a photomultiplier tube by collecting the photons in a forward direction through a 40× objective. Each oocyte was scanned across all slices in the *z*-axis using a *z*-step of 1.5 µm. For the analysis, all stacks from a single sample were processed to obtain a maximum projection image. The threshold was adjusted specifically for all samples to collect all positive signals. When necessary, LDs were separated manually in cases of obvious pixel overlay. Statistics regarding the LD’s area and number were generated automatically by LAS X software (Leica Microsystems). The LAS X software was used to apply colour-coded LD size masks to the maximum projection images for graphical observation of the distribution of LDs’ sizes in the analysed samples. The oocyte area was calculated using ImageJ (NIH, Bethesda, MD, USA) due to limitations of the LAS X software. Image acquisition and manual separation of LDs were performed in a blinded manner. A one-way ANOVA test was used based on data normality (GraphPad Prism, GraphPad Software, Boston, MA, USA).

### mRNA sequencing

Single blastocysts were snap-frozen in liquid nitrogen and stored at −80°C until further processing. Poly (A)+ RNA was isolated from individual blastocysts using the Dynabeads mRNA DIRECT Kit (Thermo Fisher Scientific, Waltham, MA, USA), and cDNA was prepared from the mRNA samples using a SMARTer Ultra Low Input RNA Kit for Illumina Sequencing (Takara Bio, Kusatsu, Japan), retaining only those samples that passed quality assessments. RNA sequencing was conducted by Novogene on the Illumina NovaSeq™ 6000 platform (Illumina, San Diego, CA, USA) (2 × 150 bp paired-end mode), generating ∼20 million read pairs per sample. The sequencing provider removed adapter sequences and low-quality reads, and the resulting clean reads were used for all downstream analyses. Following quality control, 10 samples from the YMA group and 8 samples from the AMA group were included in the analysis.

Clean reads were pseudo-aligned to the *Mus musculus* GRCm39 reference genome using Kallisto v0.46.1 (Bray *et al.*, 2016) with a transcriptome index built from ENSEMBL annotation (version 111), applying 100 bootstraps to estimate transcript abundance. The transcript-level estimates were aggregated to the gene level using tximport v1.30.0 (Soneson *et al.*, 2016) and imported into R v4.x (R Foundation for Statistical Computing, Vienna, Austria) for differential expression analysis with the DESeq2 package (Love *et al.*, 2014). Genes with a total count of fewer than 10 reads across all samples were removed prior to model fitting. Maternal age group (YMA vs AMA) was used as the only factor in the design. *P*-values were adjusted for multiple testing using the Benjamini–Hochberg procedure, and genes with an adjusted *P*-value < 0.05 and an absolute log2 fold change ≥2 were considered differentially expressed. Variance-stabilizing transformation was applied for normalization, visualization, and clustering.

Functional enrichment analysis of the differentially expressed genes was performed using GSEApy (Fang *et al.*, 2023) in Python (Python Software Foundation, Wilmington, DE, USA), querying the Enrichr platform (Chen *et al.*, 2013) against multiple databases (KEGG, Gene Ontology, Reactome, Mammalian Phenotype); only pathways or terms with Benjamini–Hochberg-adjusted *P*-value < 0.05 were retained, and top enrichments were plotted as mirrored bar charts (−log10 adjusted *P*-value). Hierarchical clustering of selected differentially expressed genes was visualized in a heatmap using the seaborn library. Data visualization and co-regulation network analysis were done in RStudio (Posit Software, Boston, MA, USA). Protein–protein network analysis for upregulated transcripts was performed in STRING (v11.5) (Szklarczyk *et al.*, 2019) using a high-confidence interaction score threshold of 0.700.

### Histology

Foetuses at embryonic Day 16 (E16) were collected and fixed for 48 h in 4% (w/v) paraformaldehyde at 4°C. Samples were then dehydrated through a graded ethanol series, cleared in a xylene mixture, and embedded in paraffin. Serial sections (7 μm thick) were cut using a microtome, mounted on glass slides, and stained with hematoxylin and eosin. Stained sections were imaged using a light microscope and analysed using ImageJ (NIH) software. Image acquisition, histological assessment, and subsequent quantifications were not performed in a blinded manner. Statistical analysis was done using a two-tailed Student’s *t*-test or Mann–Whitney’s test based on data normality; *P *< 0.05 (GraphPad Prism).

### OS marker analysis

Foetal brain and liver tissues were collected at E16 and E19 and homogenized in cold PBS supplemented with protease inhibitors. Tissue lysates were prepared by centrifugation at 2000*g* for 10 min at 4 °C, and supernatants were used for biochemical assays. Total glutathione (GSH) and superoxide dismutase (SOD) levels were quantified using colorimetric assay kits (Invitrogen), following the manufacturer’s protocols. Malondialdehyde (MDA) levels were also measured using colorimetric detection kits (Abcam, Cambridge, UK). Absorbance was recorded using a microplate reader, and concentrations were calculated from standard curves, followed by normalization to total protein content.

Statistical analysis was done using a two-tailed Student’s *t*-test or a Mann–Whitney’s test based on data normality (GraphPad Prism).

### Lipidomics

Brains were evaporated and, after resuspension in 25 µl of liquid chromatography-mass spectrometry (LC-MS) grade water, extracted using methanol (275 µl), methyl tert-butyl ether (1 ml), and 10% methanol (275 µl). An aliquot of the upper (organic) phase (400 µl) containing complex lipids was collected, evaporated, and resuspended in methanol (100 µl) prior to LC-MS analysis. Data acquisition was performed in positive ESI mode (1 µl injection) and negative ESI mode (5 µl injection). For data processing, MS-DIAL software (Tsugawa *et al.*, 2015) was used, and the lipids were annotated based on MS1 and MS/MS spectra included in the software. LC-MS Conditions: The LC-MS systems consisted of (i) a Vanquish UHPLC system (Thermo Fisher Scientific, Bremen, Germany), an Ion Max API ion source with a heated electrospray ionization (HESI-II) probe (Thermo Fisher Scientific), and a Q Exactive Plus mass spectrometer (Thermo Fisher Scientific), and (ii) a Vanquish UHPLC system (Thermo Fisher Scientific), an OptaMax NG ion source with a HESI-II probe (Thermo Fisher Scientific), and an Orbitrap Exploris 480 mass spectrometer (Thermo Fisher Scientific). The lipids were separated on an ACQUITY Premier BEH C18 column (50 mm × 2.1 mm i.d.; 1.7 μm particle size) equipped with a VanGuard FIT cartridge (5 mm × 2.1 mm i.d.; 1.7 μm particle size) (Waters, Milford, MA, USA) (Vacy *et al.*, 2024). The LC column was operated at a temperature of 65°C with a flow rate of 0.6 ml/min. LC-MS analysis was performed in positive ESI mode with the following mobile phases: (A) 60:40 acetonitrile/water with 10 mM ammonium formate and 0.1% formic acid; and (B) 90:10:0.1 isopropanol/acetonitrile/water with the same type of mobile phase modifiers. Separation was conducted using the following gradient: 0 min 15% (B); 0–1 min from 15 to 30% (B); 1–1.3 min from 30 to 48% (B); 1.3–5.5 min from 48 to 82% (B); 5.5–5.8 min from 82 to 99% (B); 5.8–6 min 99% (B); 6–6.1 min from 99 to 15% (B); 6.1–7 min 15% (B) + 1 min preinjection steps. An injection volume of 2 µl was used. The sample temperature was maintained at 4°C. The ion source and MS parameters of the Q Exactive Plus mass spectrometer were: spray voltage, +3.6 kV; sheath gas pressure, 60 arbitrary units; aux gas flow, 25 arbitrary units; sweep gas flow, 2 arbitrary units; capillary temperature, 300°C; aux gas heater temperature, 370°C; MS1 mass range, m/z 200–1700; MS1 resolving power, 35 000 FWHM (m/z 200); the number of data-dependent scans per cycle, 3; MS/MS resolving power, 17 500 FWHM (m/z 200); normalized collision energy, 20% (Menzel *et al.*, 2023). The ion source and MS parameters of the Qrbitrap Exploris 480 mass spectrometer were: spray voltage, +3.6 kV; sheath gas pressure, 60 arbitrary units; aux gas flow, 25 arbitrary units; sweep gas flow, 2 arbitrary units; capillary temperature, 300°C; aux gas heater temperature, 370°C; MS1 mass range, m/z 200–1700; MS1 resolving power, 45 000 FWHM (m/z 200); the number of data-dependent scans per cycle, 5; MS/MS resolving power, 15 000 FWHM (m/z 200); normalized collision energy, 25%. Data Processing: The LC-MS instrumental files from the untargeted lipidomic profiling were processed through MS-DIAL v. 4.9.221218 software. The lipids were annotated using an *in silico* MS/MS library in MS-DIAL and an in-house retention time–m/z library (internal standards, Chol, MG species). High-molecular-weight impurities in the mobile phase were annotated using accurate mass (MS1) and MS/MS spectra from the NIST20 library. Thermo Scientific Xcalibur Qual Browser software v. 4.1.50 was used for isotopic pattern simulation. Downstream lipidomics data analysis, multivariate calculations, and spatial modelling were performed using the MetaboAnalyst 6.0 web portal (Pang *et al.*, 2024) and RStudio (Posit Software, Boston, MA, USA), while functional pathway enrichment mapping was executed using the GO-Multiomics enrichment analysis framework (Gene Ontology Consortium *et al.*, 2023). The data pipeline was analysed using Welch’s *t*-test coupled with Benjamini–Hochberg FDR corrections to handle unequal variances.

### Proteomics

Prefrontal cortex from F3 males was weighed and ultrasonically homogenized in 20 mM HEPES (pH = 8.0). Total protein content in each sample was measured by the Bradford method. Then protein cleavage and TMT labelling were performed strictly according to the kit manufacturer’s protocol, with the aid of reagents and buffers provided by Thermo Fisher Scientific (TMT-Pro 16-plex cat. number: A44522) and Promega Corp. (Madison, WI, USA) (sequencing grade-modified trypsin V5111). After labelling, all samples were mixed to receive equal protein contents from each sample in the final mixture. Combined final sample mix was introduced into nanoLC-MS/MS system in two concentrations: 0.8 μg/injection and 1.2 μg/injection. Separation was done using Ultimate 3000 capillary chromatograph (Thermo, Bremen, Germany) equipped with 5 mm/1 mm ID trap column (Neo Trap Cartridge C18, 5 μm particle size) and analytical column: 500 mm/75 μm ID (PepMap C18, 3 μm particle size) both from Thermo Fisher Scientific. The system was connected online to Exploris 240 mass spectrometer via Nanospray Flex^TM^ ion source. Basic settings of the MS were as follows: heated capillary temperature: 275°C; scan range 300–1350 m/z; MS resolution 30 000; fragmentation mode: up to 10 MS/MS spectra per a single MS scan; MS/MS scan range: 200–2000 m/z; and MS/MS resolution: 60 000. Gradient parameters were: solvent A = water + 0.1% HCOOH (v/v) and solvent B = ACN + 0.1% HCOOH (v/v); all solvents were from Merck (Darmstadt, Germany) at the highest available purity. Optimized gradient settings were: *t* = 0, *B* = 12%; *t* = 4 min, *B* = 12%; *t* = 105 min, *B* = 27%; *t* = 107 min, *B* = 70%; *t* = 110 min, *B* = 70%; *t* = 112 min, *B* = 12%; *t* = 120 min, *B* = 12%. Flow rate was constant and equal to 300 nl/min. Raw data were converted to an *.mgf file using RawConverter software (The Scripps Research Institute, San Diego, CA, USA, ver. 1.2.0.1) and introduced into Mascot Distiller system (Matrix Science, London, UK). The Mascot Server settings applied were: Taxonomy: Mammalia; Enzyme: trypsin; max 2 missed cleavages allowed; MS tolerance: 75 ppm; MS/MS tolerance 75 ppm; #13C = 1; Quantitation = TMM Pro 16-plex; Instrument: ESI-FTICR. After calculation, a *.csv file was created and introduced into Perseus software (Tyanova *et al.*, 2016). Enrichment analysis was done in g: profiler toolset (Raudvere *et al.*, 2019), and Protein–protein network analysis was performed in STRING (v11.5) (Szklarczyk *et al.*, 2019).

## Results

### AMA is associated with an increase in oocyte lipids and alterations in chromatin modification mediators

Lipid status in AMA oocytes remains largely unknown because lipid analysis at the single oocyte level is methodologically challenging and often yields inconclusive results, especially when traditional fluorescence-based detection methods are used (Bisogno *et al.*, 2023). To characterize the extent of lipid profile alterations in AMA oocytes, multiple complementary quantitative approaches were utilized (Fig. 1).

**Figure 1.**
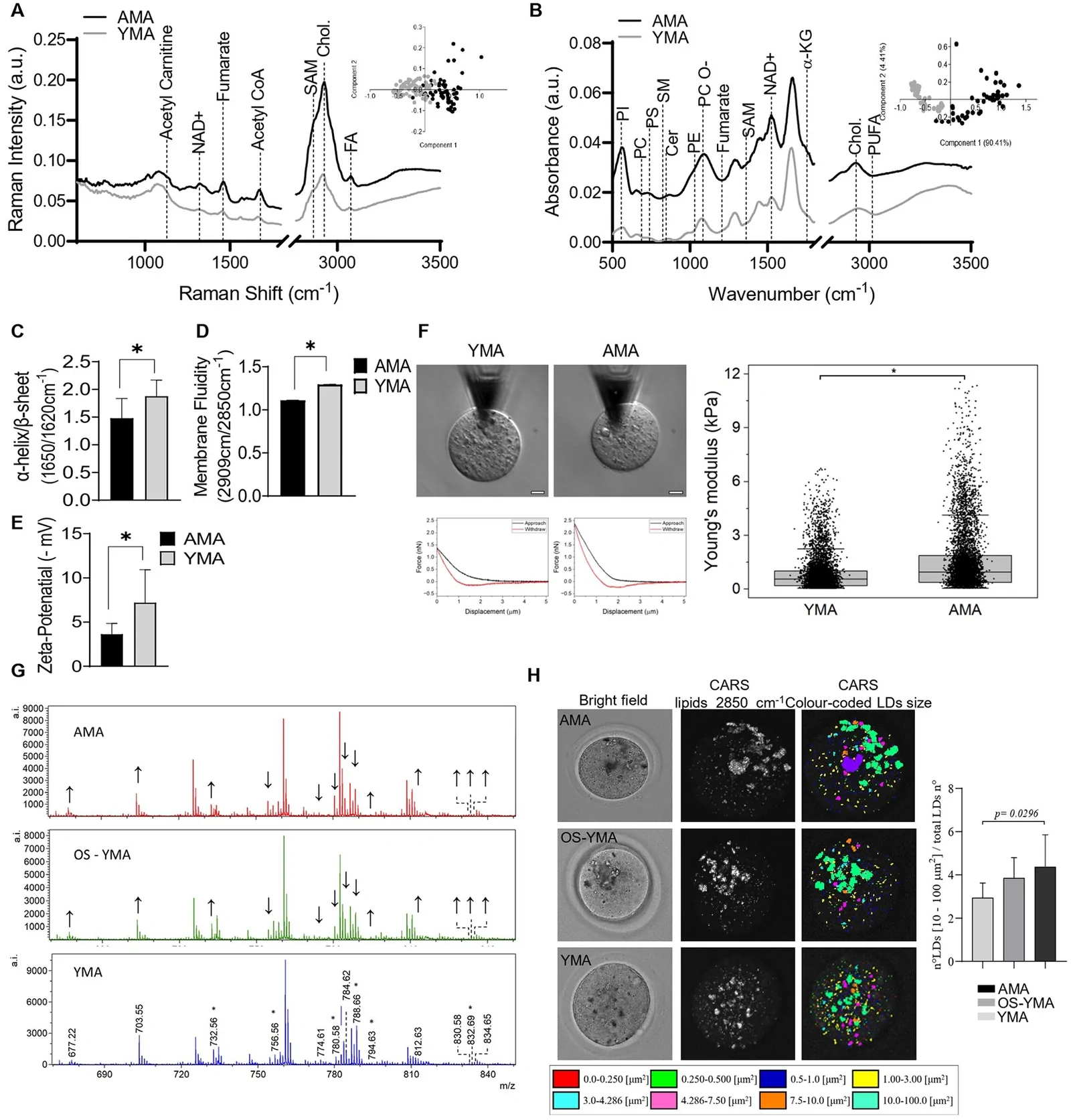
**Maternal-age-associated changes in oocyte cellular membranes and lipid abundance.** (**A**) Mean Raman spectra show high absorbance of FA, chol, and putative lipid metabolites involved in chromatin modifications: Acetyl CoA, acetylcarnitine, NAD+, fumarate, and SAM in AMA oocytes; *right corner*: PCA plot for Raman range; one-way ANOVA. (**B**) Mean FTIR spectra show higher absorbance of phospholipids (PC, PI, PS, PE, PC O–), sphingolipids (SM, Cer), cholesterol, and putative chromatin modification substrates: alpha-ketoglutarate (α-KG), fumarate, SAM, and NAD+ in AMA oocytes; *right corner*: PCA plot for FTIR range; one-way ANOVA. (**C**) Low α-helix/β-sheet ratio measured by FTIR in AMA oocytes suggests composition changes and a potential impact on protein function and aggregation; one-way ANOVA Tukey’s *post hoc* test. (**D**) and (**E**) Low membrane fluidity and mobility (ζ-potential) of AMA oocytes measured by FTIR, one-way ANOVA, and Tukey’s *post hoc* test. (**F**) Increased plasma membrane stiffness in AMA oocytes following zona pellucida removal measured by AFM analysis; *on the left*: representative optical microscopy images of YMA (left) and AMA (right) oocytes with corresponding force–displacement curves (Scale bar: 20 µm). *On the right*: box plot of the Young’s modulus values for YMA and AMA; n = 5400 data points for each group; two-tailed Student’s *t*-test; *P < 0.001. (**G**) PC changes in oocytes induced by AMA and OS. Panel shows the fragment of spectra with peaks related to PC indicated by asterisk (MALDI TOF/TOF, positive ion mode; Multivariate Partial Least Squares Regression). (**H**) CARS imaging and analysis showing increased size of LDs (10-100µm^2^) in AMA oocytes; one-way ANOVA test. **All plots** represent mean ± SD; Past.4.0 software; *P < 0.05. (A–F) refers to GV oocytes; n > 30/group, and (G and H) refers to MII oocytes; n > 20/group. α-KG, α-ketoglutarate; AFM, atomic force microscopy; AMA, advanced maternal age; CARS, coherent anti-stokes Raman spectroscopy; Cer, ceramides; Chol, cholesterol; FA, fatty acids; FTIR, fourier-transform infrared spectroscopy; LDs, lipid droplets; MALDI-TOF/TOF, matrix-assisted laser desorption/ionization tandem time-of-flight; NAD^+^, nicotinamide adenine dinucleotide; OS-YMA, oxidatively-stressed YMA; PC, phosphatidylcholine; PC O–, alkyl phosphatidylcholine; PCA, principal component analysis; PE, phosphatidylethanolamine; PI, phosphatidylinositol; PS, phosphatidylserine; PUFA, polyunsaturated fatty acids; SAM, s-adenosyl methionine; SM, sphingomyelin; YMA, young maternal age.

First, to determine whether AMA induces lipid alterations, Raman and FTIR spectroscopies were applied to single oocytes of mice at advanced age (8–16 months old, AMA) and YMA (3–4 months old). AMA oocytes exhibited increased abundance of membrane lipids such as phospholipids, sphingolipids, and Chol, and also of several metabolites that are involved in modifications of histones and DNA, such as acetyl CoA, acetylcarnitine, SAM, NAD^+^, fumarate, and α-KG (Fig. 1A and B). Because assigning specific metabolites from spectral peaks in complex biological samples can lack specificity, these molecular assignments should be considered putative. We hypothesize that elevated levels of these metabolic substrates, particularly acetyl groups, may drive genome-wide hyperacetylation, a premise that requires future validation. Acetylation neutralizes the positive charge on lysine residues in histones, weakening histone–DNA interactions and activating transcription (Celic *et al.*, 2008). In addition, a heightened abundance of FAs (especially PUFAs) is functionally linked to the reprogramming of cellular metabolism, contributing to elevated acetyl groups, histone hyperacetylation, and altered epigenetic programming (McDonnell *et al.*, 2016; Winkelkotte *et al.*, 2025). FAs also directly influence Chol signalling by modulating membrane structure and fluidity (Yang *et al.*, 2011). The abundance of Chol, together with the imbalance of several phospholipid species, such as PI, PS, PE, PC, PC O–, as well as sphingolipids (Fig. 1B), suggests that oocyte membrane structure and trafficking may be compromised in AMA oocytes (Fig. 1A and B). Changes in the lipid balance can also significantly affect the secondary structure of proteins, predominantly α-helices and β-sheets, which are critical for spanning the membrane and enabling transport and signalling. We observed changes in protein secondary structure in AMA oocytes (Fig. 1C), along with an associated low membrane fluidity (Fig. 1D), and altered membrane surface charge and stability, evidenced by low ζ-potential (Fig. 1E). These disruptions in membrane composition and physical properties directly compromise how proteins fold, integrate, and function within the bilayer, highlighting a fundamental mechanism by which AMA alters oocyte quality and potentially impacts embryonic development.

To further assess changes in the physical properties of AMA oocyte membranes, AFM was employed to analyse plasma membranes (Fig. 1F). The mean Young’s modulus value of AMA oocyte membranes was significantly higher (1.59 ± 1.29 kPa) than that of YMA oocytes (0.86 ± 0.76 kPa), indicating a stiffer and less deformable plasma membrane. This finding was made possible by the prior removal of the glycoprotein-rich zona pellucida from the oocytes and directly measuring the viscosity of the plasma membrane. To our knowledge, such an approach has not been used in AFM studies on oocytes until now. In any case, previous studies using an intact zona pellucida have demonstrated the relationship between higher rigidity of oocytes and embryos and a decreased developmental competence (Andolfi *et al.*, 2016; Yanez *et al.*, 2016). In particular, one study demonstrated the effect of postovulatory ageing of the oocyte on the mechanical properties of the zona pellucida and a relationship with embryo developmental rate (Battistella *et al.*, 2022).

Previous research by our team and others has implicated OS as a source of lipid profile changes in oocytes from older women and mice (Mihalas *et al.*, 2017; Bisogno *et al.*, 2024). To investigate this phenomenon in greater depth, the membrane lipid and LD profiles of AMA oocytes were compared with those of YMA mice exposed to OS through a 5 min treatment with tert-butyl hydroperoxide (OS-YMA). Using Matrix-Assisted Laser Desorption/Ionization—Tandem Time-of-Flight mass spectrometry (MALDI TOF/TOF MS) in a positive ion mode, both AMA and OS-YMA oocytes exhibited altered peaks of PC (a major lipid membrane component), with AMA oocytes being slightly more affected than OS-YMA (Fig. 1G; PLS analysis, Supplementary Fig. S2). Lower peaks of 756.8, 784.8, 774.8, 788.8, and 780.8 indicated the loss of some PC species in AMA and OS-YMA oocytes. Next, LD profile changes in AMA and OS-YMA oocytes were investigated using sensitive label-free Coherent anti-stokes Raman spectroscopy (CARS) imaging (Fig. 1H). An increased amount of the largest-sized LDs was discovered in AMA oocytes, most likely a sign of compensatory LPO sequestration to prevent membrane peroxidation (Gray and Woulfe, 2005; Otsuki *et al.*, 2007; Krohne *et al.*, 2010; Moreno-García *et al.*, 2018): a function that is especially important for oocytes from aged females, which face a heightened risk of oxidative damage (Titus *et al.*, 2013; Lord *et al.*, 2015; Mihalas *et al.*, 2018; Musson *et al.*, 2022). A non-significant trend towards LD accumulation was observed in OS-YMA, which may suggest that a lengthier OS exposure is required to induce significant LD changes in these oocytes.

Altogether, these findings show that the compositional and mechanical changes in AMA oocytes strongly align with female-ageing-dependent changes previously noted in human oocytes (Bisogno *et al.*, 2024). Whether these changes in lipid homeostasis persist in the embryo and offspring are the next questions to consider.

### Lipid regulation changes in AMA blastocysts

The next issue to consider was whether lipid composition changes in AMA oocytes are reflected in AMA blastocysts and whether there are any transcriptional signatures of altered membrane function robustly associated with pathway-level molecular signatures of ND conditions. RNA sequencing analysis identified a total of 172 differentially regulated genes, with 169 upregulated and 3 downregulated in AMA blastocysts (Fig. 2A). Many of these, including the strongly upregulated genes *Grin2B* and *Pcp4*, play crucial roles in synaptic plasticity (Qin *et al.*, 2005; Wei *et al.*, 2011). Their dysregulation has been linked to neurological disorders like Alzheimer’s (Jiang and Jia, 2009; Andreoli *et al.*, 2014), Huntington’s disease, and Down syndrome (Renelt *et al.*, 2014). Crucially, protein–protein interaction network modelling of these upregulated genes revealed tightly connected functional modules predominantly enriched in pathway-level molecular signatures related to synaptic vesicle cycling, glutamate receptor signalling, and GABAergic synapse pathways (Supplementary Fig. S3). Additionally, the upregulation of genes regulating lipid transport (*Cpt1b*, *Clu*, *Dgkg*) and membrane dynamics/vesicle transport (*Apba2*, *Gabra1*, *Clu*) strongly aligns with altered signalling in AMA blastocysts, consistent with previous findings of decreased exosome biosynthesis and secretion in these embryos (Bisogno *et al*., 2024). Altered vesicle transport and signalling may also reflect early molecular anomalies associated with future neurotransmission, as indicated by genes such as *Gabra1* and *Plp1*.

**Figure 2.**
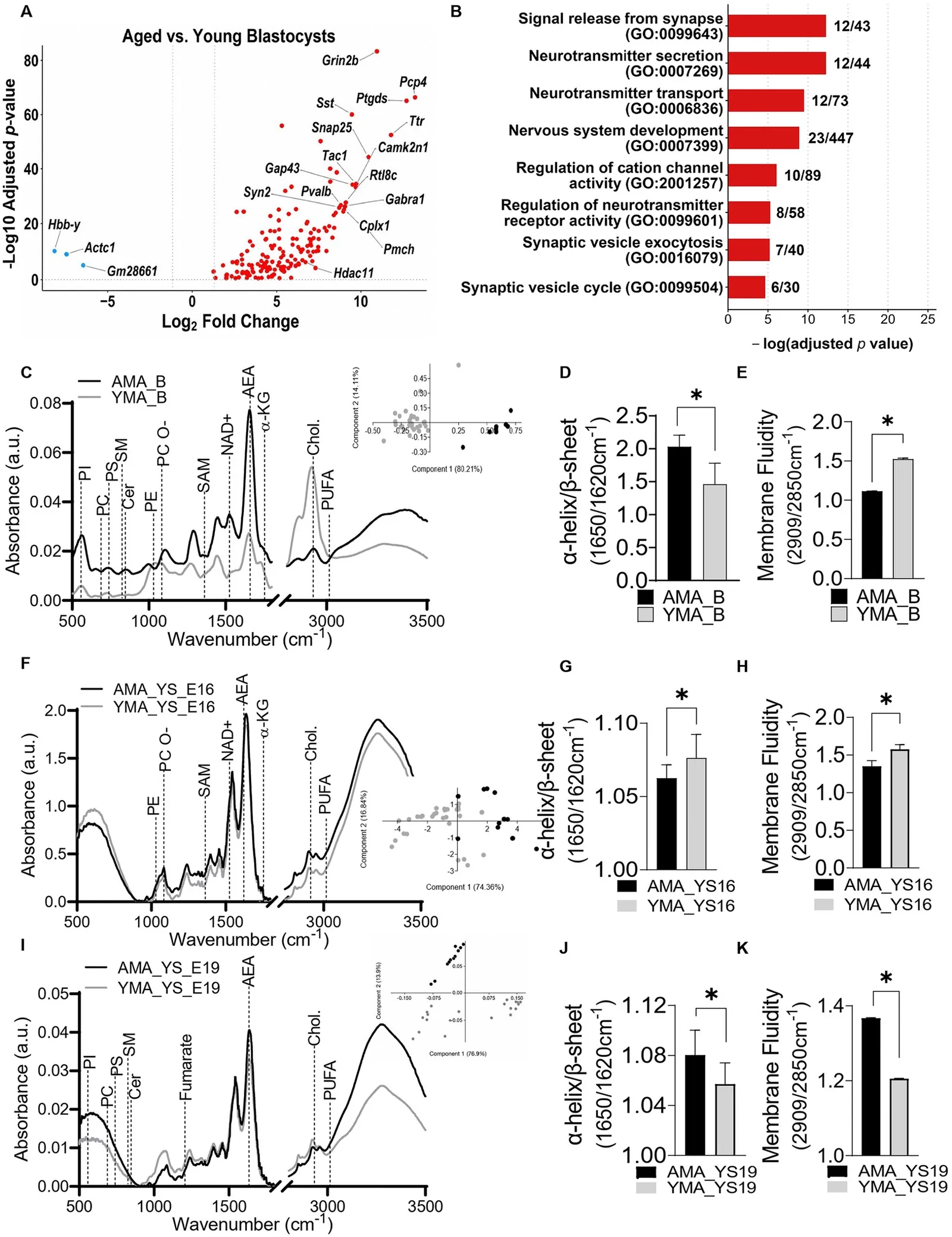
**Transcriptional deregulation of signalling and aberrant lipid profile in advanced maternal age blastocysts and yolk sacs at embryonic Days 16 and 19.** (**A**) RNAseq volcano plot visualizing upregulated genes associated with lipid signalling, synaptic pathways, and neuronal differentiation in AMA blastocysts, including the epigenetic regulator *Hdac11*. Downregulated genes in AMA blastocysts are primarily involved in oxygen transport and oxidative stress regulation. Thresholds: adjusted *P* < 0.05 (Benjamini–Hochberg false discovery rate); log2 fold change ≥ 2; n = 8–10 blastocysts/group. (**B**) Top enriched GO biological processes for all significant genes expressed in AMA blastocysts are showing enrichment in pathways related to neurotransmission. (**C**) Mean FTIR spectra showing differential absorbance of phospholipids (PI, PC, PS, PE and PC O–), sphingolipids (SM, Cer), cholesterol, PUFA, anandamide (AEA) and putative epigenetic regulators (SAM, NAD+, α-KG) in AMA blastocysts; *right corner*: PCA plot for FTIR range; AMA n = 8; YMA n = 27. (**D**) Increased α-helix and β-sheet ratio in AMA blastocysts measured by FTIR. (**E**) Low membrane fluidity of AMA blastocysts measured by FTIR. (**F**) Mean FTIR spectra showing high absorbance of phospholipids (PE and PC O–), cholesterol, PUFA, and putative epigenetic regulators: SAM, NAD+, α-KG in YS from ET AMA foetuses at E16; *right corner* PCA plot for FTIR range; AMA YS n = 11; YMA YS n = 30. (**G**) Low α-helix/β-sheet ratio in ET AMA YS at E16 measured by FTIR. (**H**) Reduced membrane fluidity in YS from AMA-derived foetuses at E16. (**I**) High absorbance of phospholipids (PI, PC, PS), sphingolipids (SM, Cer), cholesterol, PUFA, and AEA in ET AMA YS at E19; AMA n = 11; YMA n = 22. (**J**) Reduced α-helix/β-sheet ratio in ET AMA YS at E19. (**K**) Low membrane fluidity in YS from AMA-derived foetuses at E19. Panel C–K: Mean ± SD; one-way ANOVA, Tukey’s *post hoc* test; *P ≤ 0.05; Past 4.0 software. α-KG, α-ketoglutarate; AEA, anandamide; AMA, advanced maternal age; B, blastocysts; Cer, ceramides; Chol, cholesterol; E16, embryonic Day 16; E19, embryonic Day 19; FTIR, fourier-transform infrared spectroscopy; GO, gene ontology; *Hdac11*, histone deacetylase 11; NAD^+^, nicotinamide adenine dinucleotide; PC, phosphatidylcholine; PCA, principal component analysis; PC O–, alkyl phosphatidylcholine; PE, phosphatidylethanolamine; PI, phosphatidylinositol; PS = phosphatidylserine; PUFA, polyunsaturated fatty acids; SAM, s-adenosyl methionine; SM, sphingomyelin; YMA, young maternal age; YS, yolk sacs.

Most of the upregulated genes detected in AMA blastocysts are robustly associated with lipid membrane functionality and signalling but also cluster into molecular pathways governing synaptic activity, myelination, and neurotransmission pathways, all of which are essential for nervous system development (Fig. 2B). Their upregulation may represent compensatory mechanisms or altered temporal dynamics of early neurodevelopment, potentially correlating with long-term neuronal function. Notably, the chromatin-associated regulator *Hdac11* (histone deacetylase 11) was also significantly upregulated. The downregulated genes in AMA blastocysts are involved in oxygen transport, OS regulation, and lipid metabolism, such as *Actc1*, which promotes LD formation.

Next, it was observed that the higher absorbance intensities of specific lipid bands and putative chromatin modification substrates observed in AMA oocytes largely track with the spectral profiles characterized in AMA blastocysts (Fig. 2C), with the exception of peaks corresponding to PUFAs and Chol, which exhibited a lower relative absorbance intensity. Notably, AMA blastocysts displayed an increased absorbance at wavenumber regions consistent with anandamide (AEA), a known molecular marker associated with low implantation potential (Turco *et al.*, 2008). Taken together, the spectral overabundance of lipids and their metabolites, in combination with absorbance shifts reflecting altered protein secondary structure and membrane physical properties in AMA blastocysts (Fig. 2C–E), presents a strong association with a dual vulnerability: potential susceptibility to epigenetic modifications and disrupted membrane-mediated signalling. These changes strongly correlate with alterations in implantation and subsequent embryonic differentiation, with neurulation being the first key step in this developmental timeline.

### Altered lipid profiles in the YS and brain of foetal mice exhibit strong intergenerational associations with the AMA oocyte

To investigate whether altered oocyte lipids are associated with differential molecular and structural associations of ND, AMA blastocysts were transferred to young surrogate mice (ET AMA group), and YS tissue was collected for analysis at embryonic Days 16 and 19 (E16 and E19). The control group consisted of blastocysts from young females transferred to young surrogates (ET YMA). Although alterations in lipid composition and membrane properties were found to persist intergenerationally, they were slightly less pronounced in the YS than at the blastocyst stage (Fig. 2F–K), particularly near term at E19, when the levels of key metabolites return to baseline (Fig. 2I). Aberrations in the lipid homeostasis of the YS, which are robustly associated with pathways governing brain architecture, synaptogenesis, and the maintenance of neuronal networks via microglia, strongly correlate with alterations in myelination and synaptic development (Schönfeld and Reiser, 2021; Yoon *et al.*, 2022; Breach and Lenz, 2023).

The next focus was to evaluate whether the brain lipidome displays alterations in mice developed from AMA oocytes. Brains from foetal mice of ET AMA, ET YMA, and NP controls for both young and aged mothers (NP YMA; NP AMA) were collected and analysed by LC-MS. A high abundance of FA and decrease of various phospholipids in ET AMA brains were observed (Fig. 3A). FA accounted for 59.2–68.0% of increased lipid species in ET AMA in comparison to NP YMA and ET YMA groups (Supplementary Fig. S4A). Conversely, major structural and reserve lipids were downregulated in ET AMA, marked by depletions in PC (23.1–28.6%), triglycerides (TG: 15.4–23.8%), and methylated PE derivatives (PMeOH: 23.8–30.8%) (Supplementary Fig. S4A). Notably, AEA (NAE 20:4) was significantly upregulated in ET AMA brains compared to young controls, indicating a persistent multi-tissue molecular signature of AEA signalling cascades extending from the blastocyst stage.

**Figure 3.**
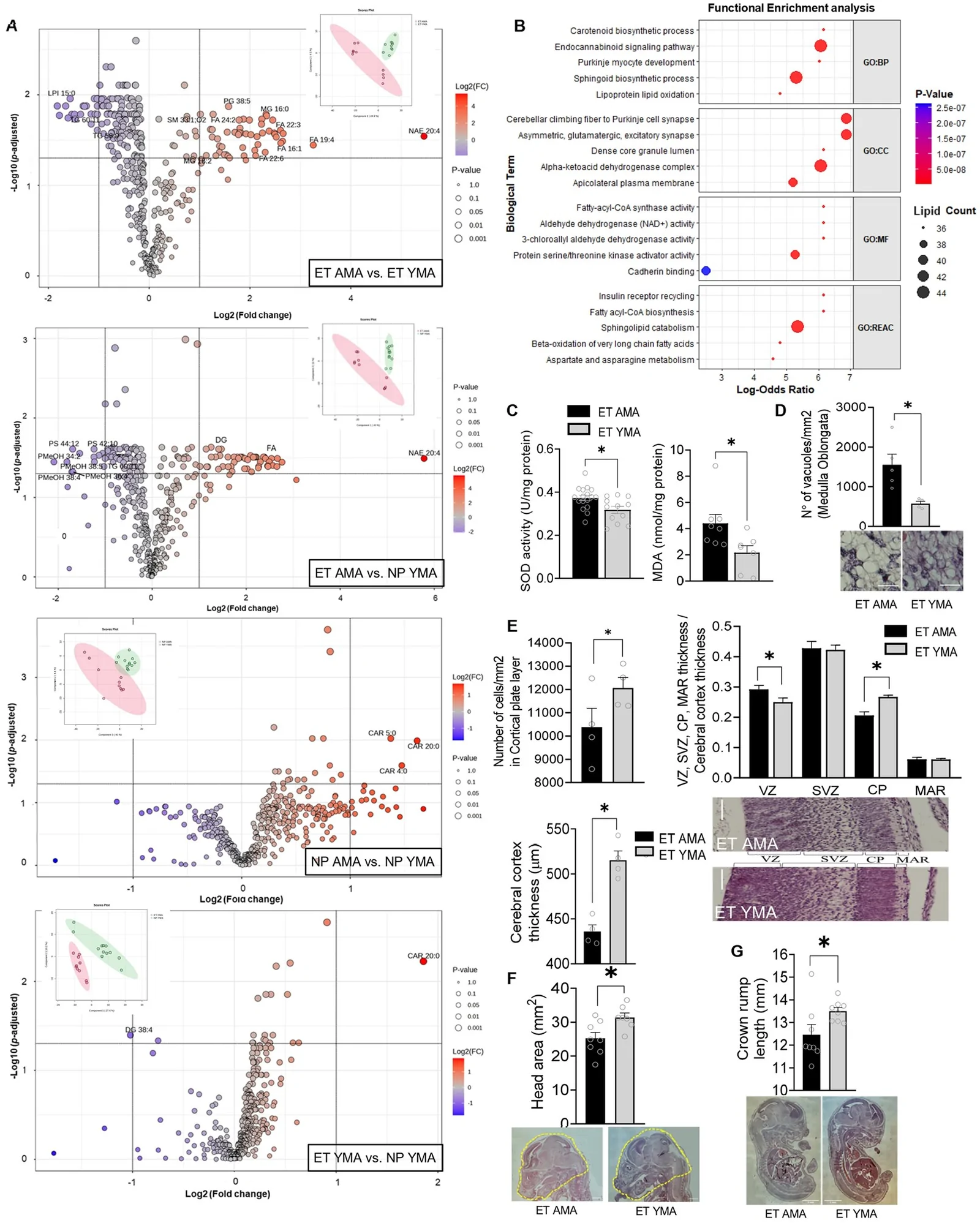
**Lipidomic profiles, oxidative markers, and morphological alterations in embryonic Day 16 foetal brain derived from advanced maternal age oocytes.** (**A**) Volcano plots displaying differentially regulated lipid species in ET AMA, ET YMA, NP AMA, and NP YMA foetal brains. ET AMA brains show abundance in FA accompanied by decrease in phospholipid levels; Welch’s *t*-test, FC > 2, FDR < 0.05; *insets*: PLS-DA shows spatial clustering and separation of groups; MetaboAnalyst 6.0. (**B**) Functional enrichment analysis mapping differentially regulated lipids to GO categories: GO: BP; GO: CC; GO: MF; GO: REAC, highlighted by log-odds ratios, *P*-values, and lipid count markers. (**C**) Brain oxidative status and increased lipid peroxidation in ET AMA-derived foetuses suggested by increased level of SOD and MDA; two-tailed Student’s *t*-test. (**D**) Increased tissue vacuolation in the medulla oblongata alongside representative histological sections demonstrating structural vacuoles; Mann–Whitney test; hematoxylin and eosin staining; mid-sagittal 7 µm sections (scale bar, 20 µm); (**E**) Upper and lower graphs and representative images on the right show decreased number of cells and less thick cerebral cortex of AMA-derived foetuses. VZ, SVZ, CP, and MAR are denoted; Mann–Whitney test; hematoxylin and eosin staining; mid-sagittal 7 µm sections (scale bar, 1 mm). (**F**) Graph and representative images of decreased head area of AMA-derived foetuses; scale bar: 1 mm. (**G**) Graphs and representative images of decreased crown rump length of AMA-derived foetuses (scale bar, 2 mm); hematoxylin and eosin staining; mid-sagittal 7 µm sections; Mann–Whitney test. Panels C–G: Data are presented as Mean ± SEM; *P < 0.05. Individual samples are indicated on bar-plots. AMA, advanced maternal age; BP, biological process; CC, cellular component; CP, cortical plate; E16, embryonic Day 16; E19, embryonic Day 19; ET, embryo transfer; FA, fatty acids; FC, fold change; FDR, false discovery rate; GO, gene ontology; MAR, marginal plate; MDA, malondialdehyde; MF, molecular function; NP, natural pregnancy; PLS-DA, partial least squares discriminant analysis; REAC, reactome pathways; SOD, superoxide dismutase; SVZ, sub-ventricular zone; VZ, ventricular zone; YMA, young maternal age.

Oxidative degradation of membrane phospholipids is known to correlate with elevated FA levels in human neural cells, tracking with a heightened susceptibility to ND conditions (Schönfeld and Reiser, 2021; Yoon *et al.*, 2022). The depletion of phospholipids, which are the source of FAs used in oxidative metabolism, is indicative of an increased level of metabolic activity. This was reflected by the top five most enriched pathways in the ET AMA brain identified through multi-databases (GO: BP, GO: CC, GO: MF, and Reactome) (Fig. 3B), describing a picture of higher membrane phospholipid turnover and lower FA breakdown (Ahmad *et al.*, 2019). In particular, long-chain and very-long-chain unsaturated FAs, such as prenateic (FA 24:2), shibic (FA 25:2), bergstromic (FA 24:4), and samuelssonic (FA 24:3) acids (Menzel *et al.*, 2023), were overabundant in ET AMA brains (Fig. 3A). Interestingly, some FA that have been associated with ageing-related degeneration, such as FA 36:6 and FA 32:5 (Liu *et al.*, 2010), were found to be upregulated in AMA brains. In NP AMA brains, where pregnancy was carried by aged mothers, an abundance of lipid metabolites including 3-hydroxyeicosanoylcarnitine (CAR 20:0), 2-Methylbutyroylcarnitine (CAR 5:0), and O-butanoyl-carnitine (CAR 4:0) were detected, which may correlate with altered epigenetic pathway activity (Conroy *et al.*, 2024).

The AMA brains were consistently grouped into two subclusters via Partial Least Squares-Discriminant Analysis, raising the possibility of sex-related distinctions within AMA groups of foetal brains, consistent with recognized sex biases in ND disorders (Zablotsky *et al.*, 2019; Breach and Lenz, 2023). However, because sample sex was not genotyped or directly assessed, no conclusions regarding sex differences can be drawn. The ET YMA versus NP YMA comparison revealed the fewest differences, indicating only a mild effect of the ET procedure on brain lipidome. Co-regulation network analysis revealed that lipid profiles of AMA groups display highly integrated correlation, indicating strong metabolic interdependence and coordinated lipid remodelling. This contrasted with the fragmented, independent modular correlation structures observed in YMA groups (Supplementary Fig. S4B).

The high FA levels observed in ET AMA brains strongly align with an OS condition, especially given the high SOD levels and increased levels of MDA (Fig. 3C), which is associated with LPO accumulation and ND disorders (Frye *et al.*, 2013). These observed peroxidative changes strongly track with alterations that affect the size and shape of the cell and overall tissue organization and thus prompted the evaluation of brain morphology (Fig. 3D–G). The medulla oblongata in ET AMA foetuses exhibited pronounced vacuolization (Fig. 3D), presenting a robust association with altered membrane lipid composition (Hanaoka *et al.*, 2024). Shifts in FAs and other membrane lipids, along with reduced cerebral cortex thickness, are robustly associated with neurodegenerative conditions and ageing (Fjell *et al.*, 2014). Impaired foetal brain growth and altered lipid levels during gestation have been linked to a higher risk of postnatal neurological conditions (Schönfeld and Reiser, 2021; Vacy *et al.*, 2024).

In ET AMA brains, the disorganized tissue structure, including the partial loss of laminar arrangement of the cells within the cortical plate (CP) and reduced CP thickness, is consistent with altered microglial clearance of damaged cellular material (notably, microglia are transiently absent in CP at E16) and strongly aligns with pathways regulating perturbed synaptogenesis (Paolicelli *et al.*, 2011; Hattori *et al.*, 2020). Taken together, the observed cell vacuolization, underdeveloped cerebral cortex, and smaller head and foetus size display a strong correlation with alterations in the physical and structural integrity of lipid membranes in the ET AMA group. Combined with disruptions in lipids and FAs (Fig. 3A and B), these alterations represent a clear pathway-level molecular signature that correlates with potential synaptic abnormalities (Madore *et al.*, 2020).

### Molecular signatures of ET AMA F3 brains reflect preceding lipid membrane anomalies in AMA oocytes and embryos

FTIR analysis revealed persistent intergenerational alterations in the absorbance intensities of membrane lipid bands (phospholipids, sphingolipids, and Chol) and membrane stability parameters in ET AMA F3 brains of adult 5-month-old male mice (Fig. 4A–D). These profiles strongly resemble the spectral alterations characterized in AMA oocytes (Fig. 1), blastocysts (Fig. 2), and foetal brains (Fig. 3), albeit less extensive. Altered membrane fluidity (Fig. 4C) and low ζ-potential (Fig. 4D) in the brains of ET AMA F3 males strongly track with alterations in tissue surface properties, which are known to correlate with pathway-level molecular signatures of synapse functionality and tissue permeability (Guy *et al.*, 2008; Walter *et al.*, 2021; Doltchinkova *et al.*, 2023). Proteomic profiling further revealed signalling pathways associated with atypical synaptic morphology and physiology, strongly aligning with pathway-level molecular signatures of altered synaptic cascades (Fig. 4E and F). Further comparison of ET AMA F3 brain proteins and AMA blastocyst transcripts revealed 31 shared elements between the two datasets, predominantly linked to pathways regulating synaptic vesicle trafficking and signalling (Fig. 4G and H). Overall, the overlap of enriched transcripts and protein pathways involved in synaptic function and architecture was robustly associated with the intergenerational persistence of similar molecular signatures across developmental windows (Fig. 4H and I).

**Figure 4.**
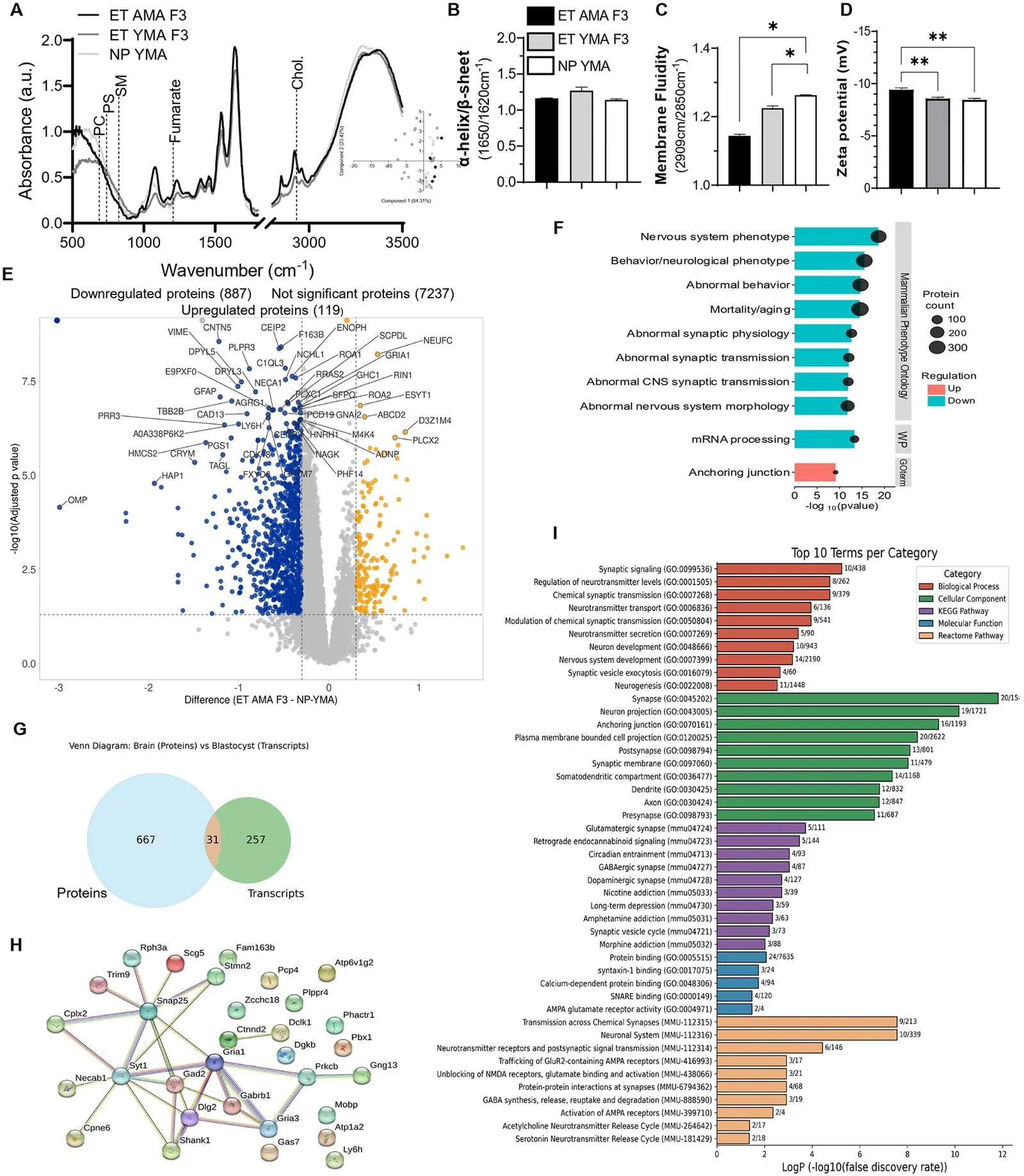
**The molecular and structural brain signatures in third-generation advanced maternal age males mirror the transcriptional profile observed in blastocysts.** (**A**) Altered absorbance of phospholipids, sphingolipids, and cholesterol in MPC of ET AMA F3 males; one-way ANOVA, *P ≤ 0.05; *right corner*: PCA plot for FTIR range. (**B**) Normal α-helix/β-sheet ratio in MPC from ET AMA F3 measured by FTIR; one-way ANOVA; *P ≤ 0.05; (**C**) Low membrane fluidity measured by FTIR. (**D**) Altered ζ-potential in MPC from ET AMA F3 suggesting physical properties consistent with altered synaptic membrane organization; one-way ANOVA, *P ≤ 0.05. (**E**) Volcano plots of detected proteins in prefrontal cortex of ET AMA F3 show deregulation of proteins involved in synaptic transmission. (**F**) The top ten most enriched pathways in prefrontal cortex of ET AMA F3. (**G**) Venn diagram displaying the overlap between unique proteins from the F3 brain dataset (blue), unique transcripts from the blastocyst dataset (green), and 31 common elements shared between both development windows (intersection). (**H**) STRING network visualization of the 31 shared transcripts/proteins illustrates functional protein–protein interactions. Node sizes and edge thicknesses correspond to interaction confidence scores, highlighting key functional clusters linked to neurodevelopment and synaptic architecture (including Shank1, Gad2, Dlg2, Gria1, and Gabrb1). (**I**) Pathway overlap analysis between ET AMA F3 brain proteins and AMA blastocyst transcripts, indicating shared pathways in synaptic signalling. Bar plot presents the top 10 enriched terms per category for the 31 shared elements. Number of samples: ET AMA F3 n = 6, ET YMA F3 n = 24, and NP YMA n = 13. AMA, advanced maternal age; Chol, cholesterol; ET, embryo transfer; FTIR, fourier-transform infrared spectroscopy; GO, gene ontology; MPC, medial prefrontal cortex; NP, natural pregnancy; SM, sphingomyelin; PC, phosphatidylcholine; PCA, principal component analysis; PS, phosphatidylserine; YMA, young maternal age.

### Lipid profile alterations in the oocytes of F3 descendants derived from AMA

To assess whether AMA-related alterations display intergenerational persistence in the ET AMA F3 germline, we analysed lipid composition in oocytes from third-generation descendants of aged females (ET AMA F3) alongside two controls: ET YMA F3 and naturally bred young females (NP YMA). A clear shift in LD distribution in ET AMA F3 oocytes was found, characterized by a reduced proportion of large LDs and an increased proportion of the smallest LD (Fig. 5A). No changes were detected in ET YMA F3 oocytes, confirming that the ET procedure itself is not robustly associated with these lipid alterations (Fig. 5A). Furthermore, the generally low phospholipid absorbance intensities, together with normal membrane fluidity and protein secondary structure (α-helix/β-sheet ratio) (Fig. 5B), indicate a partial attenuation of the oocyte lipid imbalance in the great-grand-offspring of AMA females. However, peaks corresponding to Chol and several lipid substrates associated with epigenetic regulation remain elevated. Notably, the spectral lipid profile of ET AMA F3 oocytes (Fig. 5B) closely mirrors the patterns observed in ET AMA F1 foetal brains (Fig. 3A) and ET AMA F3 adult male brains (Fig. 4A).

**Figure 5.**
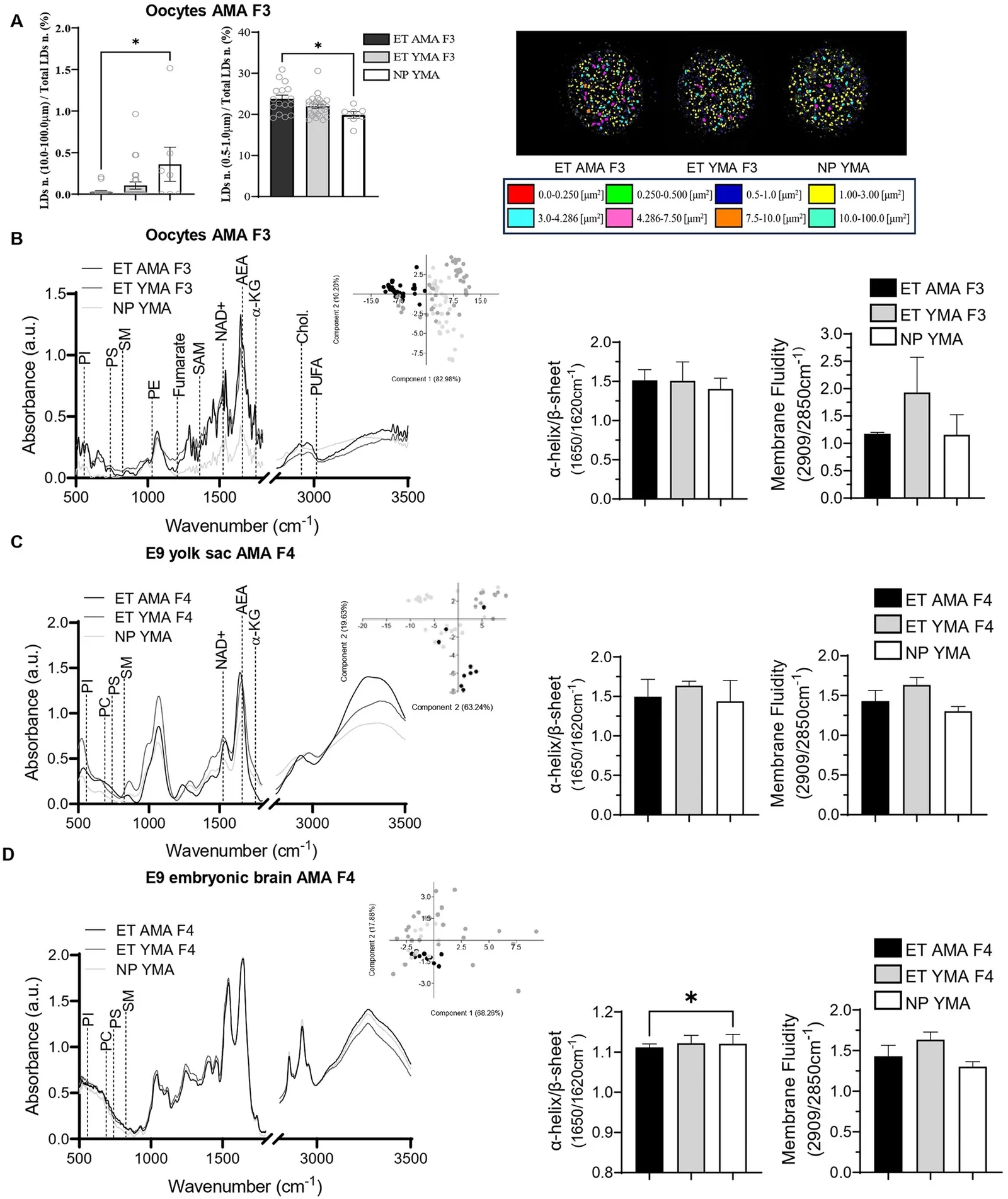
**Intergenerational persistence of lipid profile alterations in oocytes from mice derived from aged great-grandmothers and in the yolk sac of fourth-generation embryos.** (**A**) Histograms and representative CARS images show increased number of small LDs (0.5–1.0 μm^2^) and decreased number of biggest LDs (10.0–100 μm^2^) in ET AMA F3 oocytes. Colours associated with each LDs size are indicated below the images. (**B**) *From the left:* Mean FTIR spectra show altered absorbance of phospholipids (PI, PC, PS, PE), SM, Chol, PUFA, AEA, and putative epigenetic intermediates: fumarate, SAM, NAD+, α-KG in AMA in oocytes from third generation of offspring derived from ET AMA mice (ET AMA F3); Normal α-helix/β-sheet ratio and membrane fluidity in ET AMA F3 oocytes. (**C**) *From the left:* Mean FTIR spectra show altered absorbance of phospholipids (PI, PC, PS), SM, AEA, and putative epigenetic intermediates: NAD+ and α-KG in AMA in YS from ET AMA F4 collected at E9. Normal α-helix/β-sheet ratio and membrane fluidity in ET AMA F4 YS collected at E9. (**D**) *From the left:* Altered absorbance of phospholipids (PI, PC, PS) and SM in embryonic brain from ET AMA F4 at E9. Lower α-helix/β-sheet ratio measured by FTIR suggesting altered protein conformational stability and normal membrane fluidity in ET AMA F4 embryonic brain. Panels A–D: values represent mean ± SEM; one-way ANOVA test; *P ≤ 0.05; Past 4.0 software. Number of samples: E9 YS: ET AMA F4 = 15, ET YMA F4 = 9, NP YMA = 18; E9 brains: ET AMA F4 = 11, ET YMA F4 = 25, NP YMA = 10. α-KG, α-ketoglutarate; AEA, anandamide; AMA, advanced maternal age; CARS, coherent anti-stokes Raman spectroscopy; Chol, cholesterol; E9, embryonic Day 9; ET, embryo transfer; F3, third generation; F4, fourth generation; FTIR, fourier-transform infrared spectroscopy; LDs, lipid droplets; NAD^+^, nicotinamide adenine dinucleotide; NP, natural pregnancy; PI, phosphatidylinositol; PC, phosphatidylcholine; PS, phosphatidylserine; PE, phosphatidylethanolamine; PUFA, polyunsaturated fatty acids; SAM, s-adenosyl methionine; SM, sphingomyelin; YMA, young maternal age; YS, yolk sacs.

The shift toward a higher proportion of small LDs, alongside reduced phospholipid absorbance intensities and fewer large LDs in ET AMA F3 oocytes, may reflect a long-term consequence of altered metabolic programming. An alternative interpretation is that these oocytes exhibit a molecular signature consistent with the redistribution of lipid reserves to accommodate increased metabolic demand. However, such a state may be suboptimal: reduced LD content and diminished phospholipid abundance in oocytes are known to correlate with impaired signalling and lower implantation potential, as shown in our *Rab27ab* double knockout (KO) and *Gnpat* KO models (Bisogno *et al.*, 2024), and by experiments in which LDs were removed from the oocyte (Arena *et al.*, 2021). Notably, both knockout models exhibit aberrant behavioural and neuro-signalling phenotypes as well as immune alterations (Alexander *et al.*, 2017; Dorninger *et al.*, 2017). The biological significance of the distinct lipid patterns observed in oocytes from aged females in comparison to AMA F3 oocytes remains an important question for future investigation.

### Lipid profile alterations in embryonic YS and brain of ET AMA F4 embryos

After identifying lipid alterations in the oocytes of ET AMA F3 females, the next focus was whether these variations display intergenerational persistence across early embryonic windows of the fourth generation (ET AMA F4). To address this, embryonic YS and brain tissues were collected at E9, a stage at which the YS still provides the primary nutritional support to the embryo before placental function becomes established (Petry *et al*., 2026). In the YS of ET AMA F4 embryos, reduced absorbance intensities of membrane lipid bands (phospholipids and SMs), key metabolites (NAD+ and α-KG), and the wavenumber regions consistent with the lipid neurotransmitter AEA were observed (Fig. 5C). The significance of these YS alterations is highlighted by the fact that microglia, still resident in the YS at E9, will start to colonize the brain during the subsequent days of embryonic development, a process known to strongly track with pathways regulating synaptogenesis (Mauch *et al.*, 2001).

Consistent with these changes, ET AMA F4 brains exhibited lower absorbance intensities for membrane lipid bands, alongside spectral shifts reflecting alterations in protein secondary structure (Fig. 5D). Nevertheless, membrane fluidity appeared normal in both tissues, indicating that the magnitude of these fourth-generation changes remains relatively subtle (Fig. 5C and D). Overall, the lipid differences detected in these early embryonic structures of ET AMA F4 strongly parallel the spectral profile characterized in ET AMA F3 oocytes (Fig. 5B).

## Discussion

Our work demonstrates that lipid overabundance in AMA oocytes is robustly associated with molecular alterations resembling ND dysregulation. Transcriptomic and proteomic results also revealed pathway-level molecular signatures reminiscent of altered synaptic cascades and ND conditions. If confirmed by further studies assessing brain function, these findings may have important implications for the development of diagnostic and therapeutic approaches targeting oocyte quality, with the potential to reduce the risk of neurodevelopmental alterations in offspring conceived at advanced maternal age. The proposed associative framework begins with alterations in oocyte metabolism that strongly track with early embryonic pathway shifts, correlating with disruptions in lipid homeostasis, epigenetic remodelling, and the potential emergence of genetic instability in AMA conceptuses (Fig. 6).

**Figure 6.**
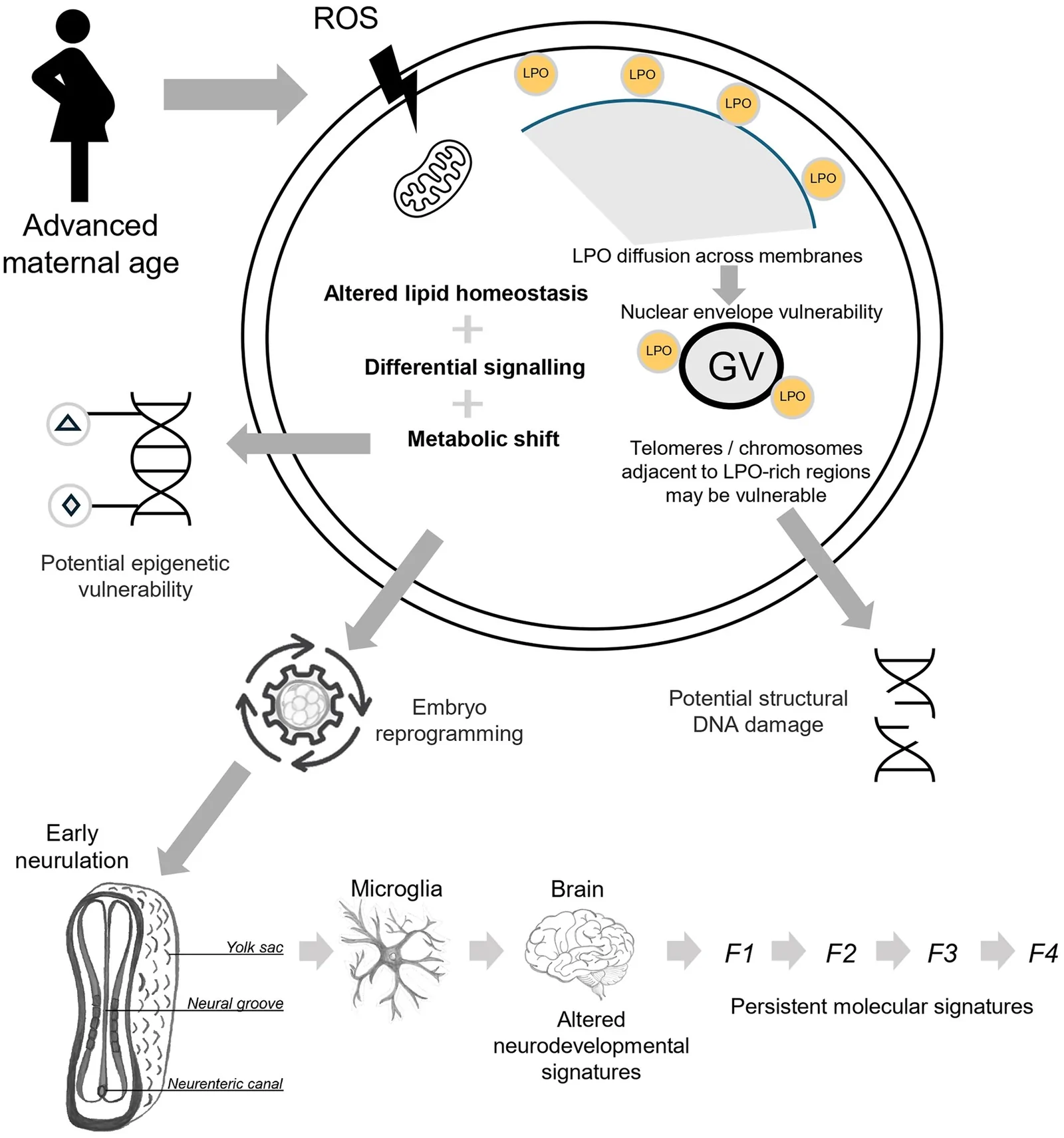
**Hypothetical framework linking age-associated oxidative changes in oocyte lipids to neurodevelopmental alterations.** Female ageing is associated with increased reactive oxygen species (ROS), altered oocyte lipid composition, and the generation of lipid peroxidation products (LPOs). Changes in structural and signalling lipids may disrupt membrane properties and cellular metabolism, including the availability of metabolites that serve as substrates or cofactors for chromatin-modifying enzymes, thereby providing a potential link between lipid metabolism and epigenetic regulation. LPOs may also diffuse within the oocyte and increase the vulnerability of chromatin located near the nuclear envelope to oxidative damage. During meiotic resumption and germinal vesicle breakdown (GVBD), dissolution of the nuclear envelope may further expose chromosomes to the LPO-rich cytoplasmic environment, potentially increasing the risk of DNA damage and chromosome-segregation errors. Independently of these possible genetic and epigenetic effects, embryos derived from advanced maternal age (AMA) oocytes must establish lipid homeostasis from fertilization onwards using maternally inherited lipid reserves. The yolk sac (YS) subsequently supports early embryonic lipid metabolism and harbours microglial precursors that later colonise the developing brain and participate in neuronal circuit formation. As placental lipid supply becomes increasingly important later in development, early disturbances in lipid composition may nevertheless persist because of the relatively slow turnover of some brain lipids. Such persistence provides a potential mechanism by which altered lipid homeostasis originating in the oocyte could remain associated with changes in neurulation, synaptic membrane organization and neurosignalling in the offspring, even in the absence of permanent genetic or epigenetic alterations.

As of yet, the significance of lipid homeostasis in the oocyte has on embryo and offspring development is not properly elucidated, especially in the context of maternal ageing. Generally, before there are any changes to more ROS-resistant cellular components, such as proteins or DNA, LPO strongly correlates with structural changes in the most sensitive cellular compartment: lipid membranes. Damaged membrane components are safely sequestered in large LDs called refractile bodies or lipofuscins, which accumulate in non-mitotic cells, such as the oocyte and the nervous system (Gray and Woulfe, 2005; Otsuki *et al.*, 2007; Krohne *et al.*, 2010; Moreno-García *et al.*, 2018). Unsurprisingly therefore, lipofuscin accumulation in the nervous system has been reported as a hallmark of ageing that correlates with neurodegenerative phenotypes (Marschallinger *et al.*, 2020). In human oocytes, the presence of large lipofuscins, which are large enough to be seen under the light microscope, is generally associated with lower developmental potential (Otsuki *et al.*, 2007). In our previous reports conducted on human and mouse oocytes, we also observed an age-related increase in oocyte LDs size (Bisogno *et al.*, 2024; Gulzar *et al.*, 2026).

After fertilization, the preimplantation embryo develops autonomously and is no longer supported by granulosa cells, leaving it without access to resources from the maternal bloodstream. This lack of external resources forces the embryo to manage disruptions in lipid homeostasis independently, tracking with metabolic shifts. Importantly, altered lipids in the embryo may still display strong associations with pathways governing neurula formation, because neurogenesis begins before the provision of maternal lipids through the placental bloodstream is established (Osso and Hughes, 2024) (Fig. 6). Subsequent restoration of lipid homeostasis in the developing brain can be rather challenging. The transport of fresh lipids to the brain is selective and limited (Hamilton *et al.*, 2007). Some lipids are characterized by a long turnover rate, often exceeding the duration of an entire pregnancy, such as lipids that compose myelin (Ando *et al.*, 2003; Osso and Hughes, 2024). In the case of short-lived organisms such as the mouse, damaged lipids may therefore persist throughout the entire lifespan (Burton, 1971). Considering that a newborn human brain is already 70% of the adult size (Clandinin *et al.*, 1997) and that lipids constitute the highest percentage of brain dry mass (∼60%) (Lawrence, 2010), it becomes evident how much ND relies on the earliest phases of life, with oocyte-derived and YS-derived lipids providing the base for the nervous system. Indeed, besides genetic damage revealed in various ND disorders in humans, non-genetic aetiologies of brain defects have been demonstrated in recent years, such as a microglial component in ASD development (Voineagu *et al.*, 2011).

Here, we show that lipid changes persist in the brain of AMA-derived prenatal mice even when gestated in the healthy uterine environment of young surrogates. Interestingly, the *in utero* environment is typically considered the important primary driver of developmental aberrancies. This is illustrated by reciprocal transfer of embryos from AMA and YMA mice, which showed that placental and foetal alterations are more pronounced in old surrogate mice carrying YMA-derived embryos (Woods *et al.*, 2017). Similarly, one study used reciprocal ovarian transplants between AMA and YMA mice to establish a maternal basis for the age-associated risk of congenital heart disease in offspring, as demonstrated by greater incidence of heart defects among pups of older mothers bearing young ovaries, rather than the inverse (Schulkey *et al.*, 2015). Clearly, the effect of the AMA uterus on embryo development is irrefutable, as shown by the lipidome changes in NP AMA embryonic brains (Fig. 3). The suboptimal pregnancy conditions arising from impaired metabolism in AMA can contribute to foetal hypoxia and OS in the developing organs, with long-term ND consequences (Krakowiak *et al.*, 2012; Comprehensive Guide to Autism, 2014). While developmental defects of AMA offspring resulting from a compromised uterine environment throughout pregnancy are not surprising, for AMA embryos transferred to young surrogate females, one would expect that lipid content and oxidative imbalance should be corrected by maternally supplied nutritional resources. However, recent reports indicate that offspring from AMA embryos transferred to young females still exhibit altered behavioural signatures (Ziȩtek and Sampino, 2023).

Apart from these direct associations of oocyte lipid oxidation with embryo development, altered lipids are known to correlate with shifts in epigenetic programming, presenting a potential pathway for lifelong and intergenerational disturbances in lipid homeostasis. Changes in availability of key metabolites (such as Acetyl-CoA and α-ketoglutarate; Etchegaray and Mostoslavsky, 2016; Fernandez-Fuente *et al.*, 2023) can modulate protein acetylation and are hypothesized to influence chromatin dysregulation, impacting further development (Donohoe and Bultman, 2012). Acetyl-CoA is essential for driving oxidative metabolism and regulating protein acetylation, a major post-translational modification. Similarly, acetylation of histones is a key epigenetic mechanism that can alter gene transcription (Etchegaray *et al.*, 2019). Additionally, changes in DNA methylation in oocytes and embryos, along with their intergenerational persistence, are crucial for correct imprinting and rely on an underlying epigenetic basis (Hua *et al.*, 2022; Potabattula *et al.*, 2022).

Lipid damage may correlate with alterations in chromosome integrity, particularly at their peripheral regions, which are most exposed to the peroxyl radicals and products of LPO present in the AMA oocyte cytoplasm. This exposure presents a potential associative pathway with chromosomal anomalies, including those associated with Down syndrome: a condition strongly linked to AMA at conception (Antonarakis *et al.*, 2020). In genetic mouse models, the presence of LPO has been implicated in pathways related to chromosomal instability (Ishihara *et al.*, 2009). Similarly, at the onset of oocyte meiotic resumption, the breakdown of the nuclear membrane may expose chromosomes to products of LPO (Fig. 6). Telomeres, which protect chromosome ends, are especially vulnerable; their shortening exhibits maternal inheritance patterns and has been robustly associated with ageing and altered lipid metabolism (Guo *et al.*, 2022; van der Spek *et al.*, 2022; Winstanley *et al.*, 2025). Apart from telomeres, other peripheral regions of chromosomes may also exhibit susceptibility, leading to segregation anomalies (Klaasen *et al.*, 2022). Overall, offspring conceived at AMA exhibit a statistically heightened risk profile for conditions that present pathway-level molecular signatures of synaptic and ND alterations, such as autism spectrum disorders, fragile X syndrome, Down syndrome, and other genetic syndromes, as well as structural defects like neural tube defects. Our hypothesis is that these risks may track with lipid-associated chromosome vulnerabilities occurring at the very start of embryogenesis.

### Limitations

Our study has several important limitations that warrant cautious interpretation of the findings. First, this research was conducted exclusively using a mouse model, which presents inherent challenges in generalizing our interpretations across all mammals. Given the distinct species-specific differences in ND organ function, baseline oocyte lipid content, and developmental kinetics, these results require careful validation before extrapolation to human clinical contexts.

Nevertheless, despite extensive morphological diversification across species, many key functions remain conserved at the molecular level. For example, RNA sequencing data performed on the YS show that its essential functions are retained across humans, mice, and birds (Cindrova-Davies *et al.*, 2017). Furthermore, the surgical ET procedure itself represents a confounding factor. The invasive nature of ET in mice has the potential to alter the embryonic microenvironment and influence metabolic programming.

Finally, our phenotypic characterization focused primarily on prenatal stages, leaving it unclear whether these foetal ND alterations ultimately manifest as synaptic dysfunction or behavioural abnormalities in adulthood. Additionally, while we documented the intergenerational persistence of these lipid-associated spectral and molecular signatures up to the fourth generation (F4), the exact underlying molecular and epigenetic mechanisms driving this long-term transmission remain uncharacterized. Investigating these postnatal outcomes and epigenetic pathways remains a key focus of our future studies.

### Concluding remarks

Little is known about the biological mechanisms underlying ND disorders. Even less is known about the involvement of lipids in their aetiology. It has been suggested that observed lipid profile changes in autistic individuals should be interpreted as co-occurring conditions, i.e. resulting from specific dietary preferences of autistic individuals (Cermak *et al.*, 2010; Luo *et al.*, 2020; Dhanasekara *et al.*, 2023; Yap *et al.*, 2023), and thus a consequence of the disorder rather than a cause. In contrast, this study identifies altered lipid homeostasis and signalling in early life as an associated background for ND signatures, where altered membrane lipids in the oocyte strongly track with molecular signatures of altered ND and synaptic pathways in the brain tissues of downstream offspring. Disrupted oocyte membranes may be functionally linked to persistent alterations in lipid composition that endure through embryonic and foetal development and may exhibit intergenerational persistence within the germline. The potential alignment of altered lipids with neurulation and synaptogenesis pathways, as well as hyperacetylation, epigenetic remodelling, and potential lipid-associated risks to chromatin integrity, may explain the complexity of changes and the multifactorial aetiology of ND disorders.

### Implications

By associating AMA oocyte lipid damage with pathway-level molecular signatures of ND alterations in offspring, our study helps to identify a potential early-life factor contributing to ND conditions. Maternal age has been rising in recent decades across the world, and the concurrent rise in ND conditions sharing synaptic pathway signatures places an additional strain on society. A higher proportion of offspring conceived at AMA may therefore correlate with broader shifts in population-level behavioural signatures in the coming decades. As such, promoting younger motherhood, as well as reducing the risk of OS through diet, supplements, and lifestyle, represent potential strategies in parenthood planning. The selection of healthy oocytes not compromised by OS may also represent an effective approach for assisted reproduction. Finally, because OS in the oocyte strongly correlates with changes in lipid homeostasis, other AMA OS-related factors that associate with ND alterations, including maternal obesity, diabetes, inflammation, drug effects, environmental pollutants, and many other conditions acting in early life, warrant exploration (Gardener *et al.*, 2009; Krakowiak *et al.*, 2012; Comprehensive Guide to Autism, 2014).

## Supplementary Material

hoag096_Supplementary_Data

## Data Availability

The transcriptomic data have been deposited in the NCBI Gene Expression Omnibus (GEO) under accession number GSE349634. Processed transcriptomic, lipidomic, and proteomic datasets, together with the shared transcript–protein analysis, are available in RODBUK at https://doi.org/10.57903/UJ/LVQ7FU. All datasets are publicly available.
