# Supplementary material for: Oocyte lipid damage from maternal ageing marks neurodevelopment in mouse offspring
Source: Hum Reprod Open. 2026 Oct 19;2026(4):hoag096. doi: 10.1093/hropen/hoag096 (PMC13760212; doi:10.1093/hropen/hoag096)
Supplement: Supplementary file 1 [file hoag096_supplementary_data.zip › HRO-26-0090-R2-SuppFigureS1-4_captions.docx]

**Supplementary Figure S1. Experimental design and multigenerational follow-up.** **A.** GV and MII oocytes and E4 blastocysts were collected from young maternal age (YMA) and advanced maternal age (AMA) mice. GV oocytes were analysed by Raman and FTIR spectroscopy, ζ-potential and atomic force microscopy (AFM); MII oocytes by MALDI-TOF/TOF MS and CARS microscopy; and E4 blastocysts by RNA-seq and FTIR. **B.** E4 blastocysts from YMA and AMA donors were transferred to young recipients (ET YMA and ET AMA), while naturally mated YMA and AMA females formed the corresponding natural-pregnancy groups (NP YMA and NP AMA). Foetal and extraembryonic tissues were collected at E16 and E19 for FTIR, lipidomic, oxidative-stress and histological analyses. **C.** ET offspring were carried to term and bred with young partners through successive generations. F3 male frontal cortex was analysed by FTIR, ζ-potential and proteomics, whereas F3 female GV oocytes were analysed by CARS and FTIR. F3 females were subsequently mated to generate F4 embryos, with E9 embryonic brain and yolk sac analysed by FTIR.

AFM, atomic force microscopy; AMA, advanced maternal age; CARS, coherent anti-Stokes Raman scattering; E4, embryonic day 4; E9, embryonic day 9; E16, embryonic day 16; E19, embryonic day 19; ET, embryo transfer; F3, third generation; F4, fourth generation; FTIR, Fourier-transform infrared spectroscopy; GV, germinal vesicle; MALDI-TOF/TOF MS, matrix-assisted laser desorption/ionisation tandem time-of-flight mass spectrometry; MII, metaphase II; NP, natural pregnancy; RNA-seq, RNA sequencing; YMA, young maternal age.

**Supplementary Figure S2.** **PLS analysis of MALDI data for AMA, YMA and OS YMA.** **A.** Predicted (x) vs. actual (y) class assignment of the groups. AMA group assigned as (-1) AMA, OS-YMA group assigned as 0, and YMA group assigned as (1). **B.** PLS Beta Regression Coefficients showing importance of variables (m/z) in the discrimination. Negative values for AMA and positive values for YMA. **C.** PLS Y Variance that is covered by a model with a number of latent variables (LV). **D.** Model error for determination of the correct number of LV.

AMA, Advanced Maternal Age; PLS, partial least squares; MALDI, matrix-assisted laser desorption/ionisation; m/z, mass-to-charge ratio; LV, latent variable; OS-YMA, Oxidatively-Stressed YMA; YMA, Young Maternal Age.

**Supplementary Figure S3. Protein-protein interaction network of upregulated genes in advanced maternal age blastocysts reveals functional hubs involved in neurosignalling.** Functional and physical interactome of upregulated genes in AMA blastocysts, generated via STRING (v11.5) using a high-confidence interaction score (0.700). The resulting network comprises 166 nodes and 138 edges (average node degree: 1.66), exhibiting significantly higher connectivity than expected by chance (PPI enrichment *p* value < 1.0e-16; 138 observed vs. 13 expected edges). This suggests a coordinated biological response among upregulated transcripts. Functional modules were identified through MCL clustering (inflation parameter: 3.0), with node colours representing distinct biological clusters. The five primary functional hubs (Clusters 1–5) are predominantly involved in synaptic vesicle cycling, glutamate receptor signalling (long-term potentiation), and GABAergic synapse pathways. Edge thickness indicates the strength of data support, and disconnected nodes were omitted to highlight primary functional hubs. The specific gene compositions and enriched biological pathways for the five primary functional modules are detailed in Table.

AMA, Advanced Maternal Age; GABA, Gamma-Aminobutyric Acid; MCL, Markov Cluster Algorithm; PPI, Protein-Protein Interaction.

**Supplementary Figure S4. Compositional analysis and co-regulation networks of differentially-regulated lipid classes across experimental groups.**
**A**. Donut plots displaying the percentage distribution of significantly upregulated (left) and downregulated (right) lipid classes for the ET AMA vs. ET YMA comparison (top) and the ET AMA vs. NP YMA comparison (bottom); **B**. Hierarchical clustering and co-regulation heatmaps mapping Pearson correlation coefficients among lipid classes across NP-AMA (top left), ET-AMA (top right), ET-YMA (bottom left), and NP-YMA (bottom right). Notably, the lipid profiles of NP-AMA and ET-AMA cohorts display highly integrated, blocks of positive and negative correlation, indicating strong metabolic interdependence and coordinated lipid remodelling. This contrasts with the fragmented, independent modular correlation structures observed in the young maternal age controls (ET-YMA and NP-YMA). Analysis performed using RStudio.

AMA, Advanced Maternal Age; CAR, Acylcarnitine; CE, Cholesteryl Esters; CL, Cardiolipin; DG, Diglycerides; ET, Embryo Transfer; FA, Fatty Acids; GM3, Ganglioside; LPC, Lysophosphatidylcholine; LPE, Lysophosphatidylethanolamine; LPG, Lysophosphatidylglycerol; LPI, Lysophosphatidylinositol; MG, Monoglycerides; MGDG, Monogalactosyldiacylglycerol; NAE, N-acylethanolamines; NP, Natural Pregnancy; PC, Phosphatidylcholine; PE, Phosphatidylethanolamine; PG, Phosphatidylglycerol; PI, Phosphatidylinositol; PM / PMeOH, Methylated phosphatidylethanolamine; PS, Phosphatidylserine; SH, Sulfatides; SM, Sphingomyelin; SPB, Sphingoid Bases; ST, Sterols; TG, Triglycerides; YMA,Young Maternal Age.
